# Treatment of severe acute respiratory syndrome (SARS), Middle East respiratory syndrome (MERS), and coronavirus disease 2019 (COVID-19): a systematic review of *in vitro*, *in vivo*, and clinical trials

**DOI:** 10.7150/thno.48342

**Published:** 2021-01-01

**Authors:** Young Joo Han, Keum Hwa Lee, Sojung Yoon, Seoung Wan Nam, Seohyun Ryu, Dawon Seong, Jae Seok Kim, Jun Young Lee, Jae Won Yang, Jinhee Lee, Ai Koyanagi, Sung Hwi Hong, Elena Dragioti, Joaquim Radua, Lee Smith, Hans Oh, Ramy Abou Ghayda, Andreas Kronbichler, Maria Effenberger, Daniela Kresse, Sara Denicolò, Woosun Kang, Louis Jacob, Hanwul Shin, Jae Il Shin

**Affiliations:** 1 Department of Pediatrics, Samsung Changwon Hospital, Sungkyunkwan University School of Medicine, Changwon, Republic of Korea.; 2 Department of Pediatrics, Yonsei University College of Medicine, Seoul, Republic of Korea.; 3 Yonsei University College of Medicine, Seoul, Republic of Korea.; 4 Department of Rheumatology, Yonsei University Wonju College of Medicine, Wonju, Republic of Korea.; 5 Department of Nephrology, Yonsei University Wonju College of Medicine, Wonju, Republic of Korea.; 6 Department of Psychiatry, Yonsei University Wonju College of Medicine, Wonju, Republic of Korea.; 7 Research and development unit, Parc Sanitari Sant Joan de Déu/CIBERSAM, Universitat de Barcelona, Fundació Sant Joan de Déu, Sant Boi de Llobregat, Barcelona, Spain.; 8 ICREA, Pg. Lluis Companys 23, 08010, Barcelona, Spain.; 9 Department of Global Health and Population, Harvard T.H. Chan School of Public Health, 677 Huntington Avenue, Boston, USA.; 10 Pain and Rehabilitation Centre, and Department of Health, Medicine and Caring Sciences, Linköping University, Linköping, Sweden.; 11 Institut d'Investigacions Biomèdiques August Pi i Sunyer (IDIBAPS) and Mental Health Research Networking Center (CIBERSAM), Barcelona, Spain.; 12 Department of Psychosis Studies, Institute of Psychiatry, Psychology and Neuroscience, King's College London, London, UK.; 13 Centre for Psychiatric Research, Department of Clinical Neuroscience, Karolinska Institutet, Stockholm, Sweden.; 14 The Cambridge Centre for Sport and Exercise Sciences, Anglia Ruskin University, Cambridge, UK.; 15 School of Social Work, University of Southern California, CA, USA.; 16 Division of Urology, Brigham and Women's Hospital and Harvard Medical School, Boston, MA, USA.; 17 Department of Internal Medicine IV (Nephrology and Hypertension), Medical University Innsbruck, Innsbruck, Austria.; 18 Department of Internal Medicine I (Gastroenterology, Hepatology, Endocrinology & Metabolism), Medical University Innsbruck, Innsbruck, Austria.; 19 Department of Internal Medicine, St. Johann County Hospital, St. Johann in Tirol, Austria.; 20 Department of Internal Medicine, University of Illinois College of Medicine at Peoria, Peoria, IL, USA.; 21 Faculty of Medicine, University of Versailles Saint-Quentin-en-Yvelines, Montigny-le-Bretonneux, France.

**Keywords:** COVID-19, therapeutic agent, SARS, MERS, mortality, coronavirus

## Abstract

**Rationale:** Coronavirus disease 2019 (COVID-19) has spread worldwide and poses a threat to humanity. However, no specific therapy has been established for this disease yet. We conducted a systematic review to highlight therapeutic agents that might be effective in treating COVID-19.

**Methods:** We searched Medline, Medrxiv.org, and reference lists of relevant publications to identify articles of *in vitro*, *in vivo*, and clinical studies on treatments for severe acute respiratory syndrome (SARS), Middle East respiratory syndrome (MERS), and COVID-19 published in English until the last update on October 11, 2020.

**Results:** We included 36 studies on SARS, 30 studies on MERS, and 10 meta-analyses on SARS and MERS in this study. Through 12,200 title and 830 full-text screenings for COVID-19, eight *in vitro* studies, 46 randomized controlled trials (RCTs) on 6,886 patients, and 29 meta-analyses were obtained and investigated. There was no therapeutic agent that consistently resulted in positive outcomes across SARS, MERS, and COVID-19. Remdesivir showed a therapeutic effect for COVID-19 in two RCTs involving the largest number of total participants (n = 1,461). Other therapies that showed an effect in at least two RCTs for COVID-19 were sofosbuvir/daclatasvir (n = 114), colchicine (n = 140), IFN-β1b (n = 193), and convalescent plasma therapy (n = 126).

**Conclusions:** This review provides information to help establish treatment and research directions for COVID-19 based on currently available evidence. Further RCTs are required.

## Introduction

Coronavirus disease 2019 (COVID-19) refers to a respiratory syndrome caused by infection with severe acute respiratory syndrome coronavirus 2 (SARS-CoV-2), an RNA virus belonging to the Coronaviridae family. Ever since the disease was first reported in Wuhan, China in December 2019, it has spread rapidly around the world. On October 28, 2020, a total of 43,766,712 SARS-CoV-2 cases were reported worldwide, of which 1,163,459 died 1. Clinical manifestations range from being asymptomatic to pneumonia and acute respiratory distress syndrome (ARDS). Although estimations of case-fatality rate are different for COVID-19, there appears to be a high rate of a severe disease course or death, mainly in patients with advanced age or underlying diseases 2, 3. Current case fatality rates are 2.2% in Africa, 3.2% in Americas, 2.5% in Eastern Mediterranean Region, 3.2% in Europe, 1.6% in South-East Asia, and 2.1% in Western Pacific Region 1, whereas the case fatality rate of SARS and Middle East respiratory syndrome (MERS), which are coronavirus respiratory syndromes similar to COVID-19 were 11% 4 and 34% 5, respectively.

There are currently no specific established treatments for COVID-19. Since the outbreak of COVID-19, numerous studies have been conducted during the past months; however, it is difficult to extract information from these extensive studies, synthesize the results, and apply them in practice. In fact, it would be almost impossible for front-line medical practitioners to be able to absorb the considerable number of reports being released on a daily basis and immediately translate the findings into practice during this medical crisis.

For this reason, we summarized the *in vivo*, *in vitro*, and clinical research results related to potential therapies of COVID-19 and further integrated the results with previously reported results from SARS and MERS. We aimed to provide useful information for the establishment of treatment and research directions for COVID-19.

## Methods

### Literature search strategy and study selection

We adhered to the Preferred Reporting Items for Systematic Reviews and Meta-Analyses (PRISMA) statement. Two investigators (YJH and JIS) manually searched Medline for literature regarding therapeutics for SARS, MERS, and COVID-19. Only publications in English were included, with the exception of an individual study used within a meta-analysis.

**Figure 1:**
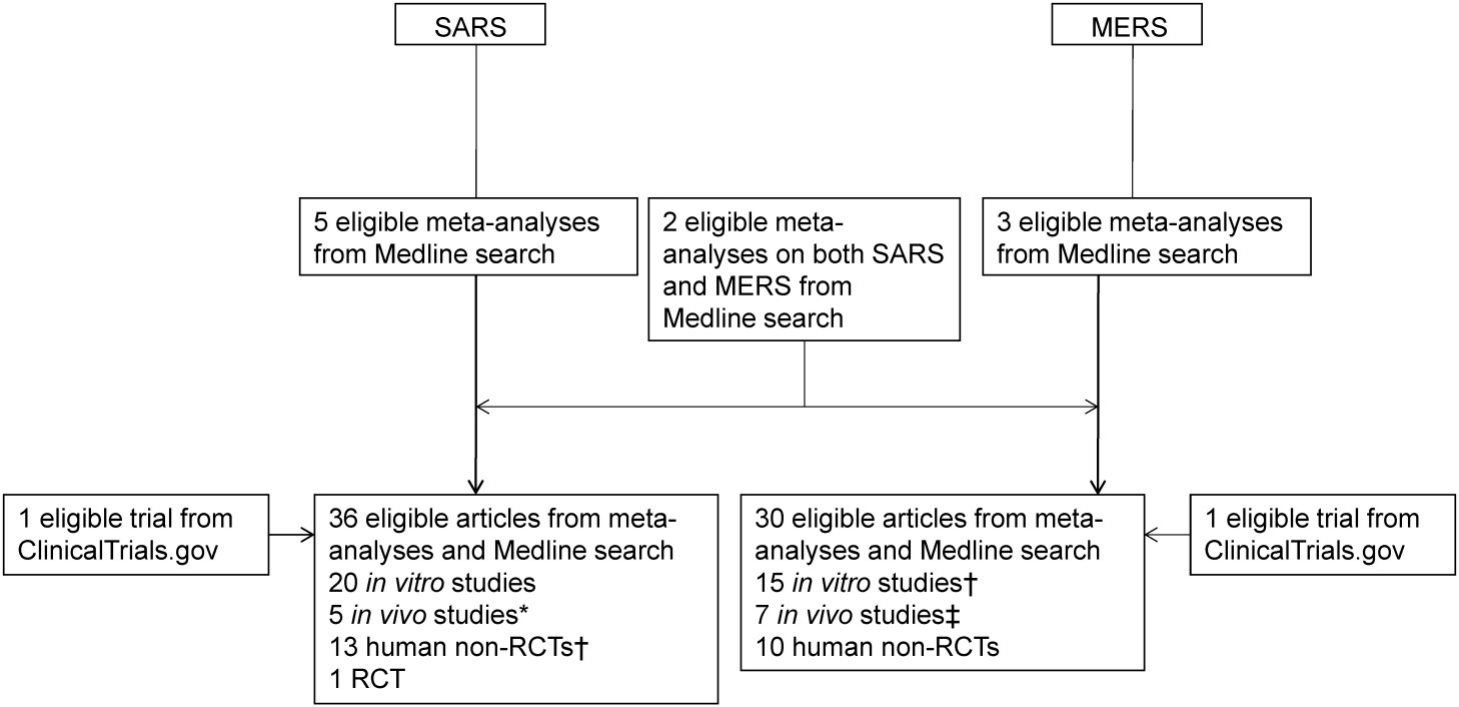
** Flowchart of article selection process for SARS and MERS.** SARS: severe acute respiratory syndrome; MERS: Middle East respiratory syndrome; RCT: randomized controlled trial. *Two overlapped with *in vitro* studies on SARS; †One overlapped with an *in vitro* study on SARS each; ‡One overlapped with *in vitro* studies on MERS.

In order to complete this review in a timely manner during this pandemic, we first searched the meta-analyses or systematic reviews on SARS and MERS from inception to March 31, 2020 using the following search terms (“severe acute respiratory syndrome”, “SARS”, “Middle East respiratory syndrome”, or “MERS”) and (“meta”[title] or “systematic” [title]). After reading the full-text of articles obtained as a result of this search, we also investigated the *in vitro*, *in vivo*, and human studies on therapeutics of SARS or MERS that were included in them. Next, we conducted an additional search using the following search terms for the parts that were considered to be necessary for replenishment: [(“severe acute respiratory syndrome” or “SARS”) and (“remdesivir”, “nelfinavir”, “interferon beta”, or “chloroquine”)] or [(“Middle East respiratory syndrome” or “MERS”) and (“remdesivir”, “lopinavir”, “ritonavir”, “interferon alpha”, “interferon beta”, “convalescent plasma”, “chloroquine”, or “corticosteroid”)] (**Figure 1**).

**Figure 2:**
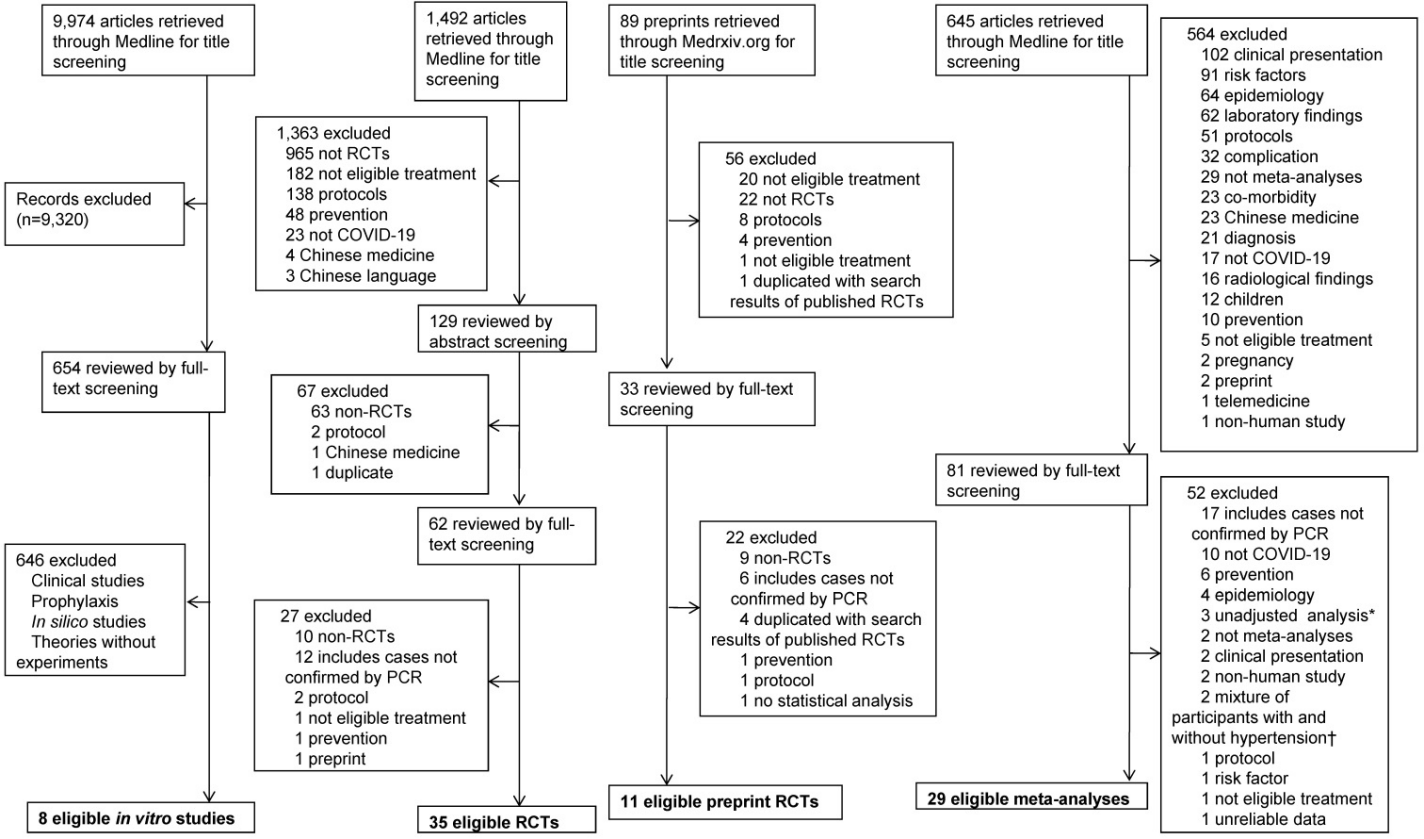
** Flowchart of article selection process for COVID-19.** COVID-19: coronavirus disease 2019; PCR: polymerase chain reaction; RCT: randomized controlled trial. *Including non-RCTs on corticosteroid therapy for patients with various severity of COVID-19. †Studies on angiotensin-converting enzyme inhibitor or angiotensin receptor blocker.

Moreover, in order to search for studies on COVID-19, a search was performed through the following search algorithm until the last update on May 7, 2020: ((wuhan[All Fields] and (“coronavirus”[MeSH Terms] or “coronavirus”[All Fields])) and 2019/12[PDAT]: 2030[PDAT]) or 2019-nCoV[All Fields] or 2019nCoV[All Fields] or COVID-19[All Fields] or SARS-CoV-2[All Fields]**.** In addition, a search for randomized controlled trials (RCTs) on COVID-19 was also performed using the following search terms until the last update on October 9, 2020: ((((wuhan[All Fields] and (“coronavirus”[MeSH Terms] or “coronavirus” [All Fields])) and 2019/12 [PDAT]: 2030[PDAT]) or 2019-nCoV[All Fields] or 2019nCoV [All Fields] or COVID-19[All Fields] or SARS-CoV-2[All Fields]) and (random [Title/Abstract] or randomization [Title/Abstract] or randomized [Title/Abstract] or randomized [Title/Abstract] or trial[Title]). To include a more sufficient amount of RCTs, a search for preprint RCTs through the database of Medrxiv.org was performed by conditions that include the following search terms in the titles until the last update on October 11, 2020: [“COVID” and (“random”, “controlled”, or “trial”)] or [“coronavirus” and (“random”, “controlled”, or “trial”)]or [“cov” and (“random”, “controlled”, or “trial”)]. A search for meta-analyses of treatment for COVID-19 was performed using the following search terms until the last update on October 11, 2020: ((((wuhan[All Fields] and (“coronavirus”[MeSH Terms] or “coronavirus”[All Fields])) and 2019/12[PDAT]: 2030[PDAT]) or 2019-nCoV[All Fields] or 2019nCoV[All Fields] or COVID-19[All Fields] or SARS-CoV-2[All Fields]) and (meta[Title]) (**Figure 2**).

### Eligibility criteria

Two investigators (YJH and JIS) identified the eligible studies by screening the titles and abstracts independently. Any disagreement was resolved by discussion and consensus among review authors. For non-human research, eligibility criteria for inclusion were (1) studies on SARS-CoV, MERS-CoV, or SARS-CoV-2 and (2) studies in which inoculation of virus preceded administration of therapeutic agents. For human research, eligibility criteria were organized in accordance with the Participants, Interventions, Comparisons, and Outcomes (PICO) reporting structure.

### Participants

We included studies on individuals with SARS, MERS, or COVID-19 who were diagnosed by validated methods using real time reverse transcription polymerase chain reaction (PCR) 6. We excluded studies that were performed exclusively in children. According to the 7th edition of the Chinese clinical guidance for COVID-19 pneumonia, treatment with corticosteroids, tocilizumab, or convalescent plasma was recommended for patients with severe or progressive COVID-19 7. Therefore, when a non-RCT was included in a meta-analysis and targeted any of these treatment forms for patients with different severity of COVID-19, only the study analyzing the results of multivariate analysis conducted in the original research was included. In the case of meta-analyses on angiotensin-converting enzyme inhibitor (ACEI) or angiotensin receptor blocker (ARB) for COVID-19, studies that did not include participants selectively according to the presence of hypertension were excluded because it was thought that a mixture of participants with and without hypertension would affect the treatment outcome.

### Interventions

We considered the pharmacological, immunological, or miscellaneous therapies administered after the onset of infection. Multiple therapeutic agents in combination were also included. Types of respiratory support, mechanical ventilation (MV) strategy, extracorporeal therapy, and radiation therapy were not target interventions in this study. The exclusion criteria were studies on (1) immunization or chemoprophylaxis, (2) Chinese medicine, or (3) other topics, such as epidemiology, without dealing with therapeutic interventions. We also excluded non-RCTs that did not specify the number of patients in the intervention group.

### Comparisons

Control interventions relevant to the general treatment of respiratory infection (*e.g*., placebo or usual medications) or other therapeutic agents that could be candidates for the study intervention were included.

### Outcomes

Studies reporting on mortality, intensive care unit (ICU) admission, disease progression, discharge rates, or improvement in the chest radiograph in the intervention/entire patient group or control group were included.

### Study design

Because RCTs on SARS or MERS performed to date were not sufficient, any RCT, study in prospective or retrospective cohort design, case-control design, or case series published as an article in a scientific journal were eligible. In the case of non-RCTs, studies with a total of 10 or more patients were included, except for relatively rare treatment forms that had not been administered in dozens of patients to date. For COVID-19, only RCTs were eligible except of studies included in a meta-analysis.

### Data extraction

Two investigators (YJH and JIS) collected information on the total number of patients and the number of patients in the intervention group, time range of enrollment or at the time of diagnosis or hospitalization, intervention and control therapy used in the study, and the outcome among the intervention and the control group.

### Classification of studies and interpretation of results

In order to interpret the results of *in vitro* studies, 50% maximal effective concentration (EC_50_) less than 10 μM or selectivity index (SI) greater than 10 was set as a criterion for determining whether a particular drug has therapeutic potential against the virus of interest.

The results of RCTs and meta-analyses were categorized as follows, depending on whether the therapeutic agent was effective against COVID-19.

Effective: The treatment group showed superior results for major outcomes (mortality, ICU admission, disease progression, discharge, clinical improvement, or improvement in the chest radiograph) with a statistical significance (*P* < 0.05).

Possible effect: The major outcome of the treatment group was not significantly worse (*P* > 0.05), and the results for other outcomes other than the major outcome was superior in the treatment group with a statistical significance (*P* < 0.05).

Not effective: The results for any outcome did not show a significant difference between the treatment and the control group (*P* > 0.05).

Possible harm: The treatment group did not show statistically superior results for major outcomes (*P* > 0.05), and the results for other outcomes were worse with a statistical significance (*P* < 0.05).

Harmful: The treatment group showed statistically inferior results for the major outcome (*P* < 0.05).

## Results

### Systematic search results

Through Medline search, a total of 10 meta-analyses on SARS (n = 5) 8-12, MERS (n = 3) 13-15, and both (n = 2) 16, 17 were obtained and investigated. After investigating the original texts of *in vitro*, *in vivo*, and clinical studies cited in these meta-analyses, an additional Medline search was performed when the clinical study obtained by the search seemed to be insufficient for therapeutic agents that showed positive results from *in vitro* or *in vivo* studies. Through this process, 36 and 30 eligible articles on SARS and MERS were obtained, respectively: 20 *in vitro*, five *in vivo* studies (two overlapping with *in vitro* studies on SARS), 13 human non-RCTs (one overlapping with an *in vitro* study on SARS), and one RCT on SARS; and 15 *in vitro* (one overlapping with an *in vitro* study on SARS), seven *in vivo* studies (one overlapping with *in vitro* studies on MERS), and 10 human non-RCTs on MERS. In addition, as a result of searching the database of Clinicaltrials.gov, we identified one RCT on SARS that was not completed after registration, and one on MERS. Of these, an RCT of lopinavir/ritonavir plus ribavirin in the treatment of SARS 18 had not yet started recruiting participants since it was registered in December 2007, and the current status was unknown; and another RCT of lopinavir/ritonavir and interferon (IFN)-β1b in the treatment of MERS 19 was completed on May 20, 2020 **(Figure 1)**.

A total of 12,200 articles on COVID-19 were identified through a Medline and Medrxiv.org search. After full-text screening of 830 articles, 83 eligible articles on COVID-19 were obtained: eight *in vitro* studies, 46 RCTs on 6,886 patients, and 29 meta-analyses **(Figure 2)**.

The research results for SARS and MERS for each therapeutic agent are described in **Table 1**, and the research results for COVID-19 are described in **Table 2, 3 & Table 4**, and **Table S1**.

### Antiviral agents

#### Remdesivir

Remdesivir showed effects in multiple non-human studies on SARS or MERS (**Table 1**), and in one *in vitro* study on COVID-19 (**Table 2**). Four RCTs on remdesivir for COVID-19 have been published to date. One of them was a large-scale RCT, with 538 and 521 participants in the treatment and the control group, respectively, and remdesivir was administered to the treatment group for 10 days. The time to recovery was shorter in the treatment group compared to the control group {11 [95% confidence interval (CI) 9-12] *vs.* 15 13-19 days; relative risk (RR) for recovery 1.32, 95% CI 1.12 to 1.55; *P* < 0.0001} and the odds ratio (OR) for the improvement of the ordinal score on day 15 was 1.50 (95% CI 1.18 to 1.91, *P* = 0.001). There was no significant difference in the 14-day mortality rate between the two groups. However, when compared among the participants with a baseline ordinal score of 5 requiring oxygen supplementation, the 14-day mortality rate of the treatment group was significantly lower (4 out of 222 [2%] *vs.* 19 out of 199 [10%]; hazard ratio [HR] 0.22, 95% CI 0.08 to 0.58) 20. In another RCT on severe COVID-19, the 28-day mortality rate did not differ between remdesivir-treated patients and controls 21 (**Table 3**). According to a meta-analysis for these two RCTs 20, 21, the RR for clinical recovery was 1.17 (95% CI 1.07 to 1.29) 22 (**Table 4**).

The other two RCTs for COVID-19 were performed with different administration periods of remdesivir, five and ten days, respectively. Among them, the 5-day treatment group showed better clinical status distribution on the 7-category ordinal scale on day 11 (OR 1.65, 95% CI 1.09 to 2.48) in one RCT for moderate COVID-19 23. In another RCT for severe COVID-19, the incidence of serious adverse events was lower in the 5-day treatment group than in the 10-day treatment group (42 out of 200 [21%] *vs.* 68 out of 197 [35%]; difference 10.8%, 95% CI 2.4% to 19.2%) 24 (**Table 3**). In a meta-analysis involving one of these RCTs 24 and another unreported RCT 25, the OR for clinical recovery in the 5-day course of treatment was 1.33 (95% CI 1.01 to 1.76) compared to the 10-day course of treatment 26 (**Table 4**).

#### Sofosbuvir and daclatasvir

A combination of sofosbuvir/daclatasvir showed an effect in two RCTs on COVID-19 which were conducted in Iran. In one RCT, the cumulative incidence of hospital discharge was higher (*P* = 0.041) and the duration of hospitalization was shorter (6 [interquartile range (IQR) 4-8] *vs.* 8 days 5-13; *P* = 0.029) in the treatment compared to the control group 27. In another RCT, the cumulative incidence of recovery was higher in the treatment compared to the control group (*P* = 0.033) 28 (**Table 3**).

#### Favipiravir

The results of two *in vitro* studies on favipiravir for COVID-19 were unfavorable 29, 30. However, in a Russian RCT on favipiravir for moderate COVID-19, the rate of negative results of virus PCR on day 5 was higher in the treatment than in the control group (25 out of 40 [63%] *vs.* 6 out of 20 [30%]; *P* = 0.018) 31. In another RCT on mild COVID-19, the hospital discharge rate of participants who received favipiravir from the first day of enrollment was higher than that of participants who received favipiravir starting from one week after enrollment (HR 2.68, 95% CI 1.67 to 4.29) 32 (**Table 3**).

#### Umifenovir

Umifenovir showed an effect in an *in vitro* study on COVID-19 33 (**Table 2**). In an RCT comparing a combination of umifenovir and lopinavir/ritonavir with standard treatment, the group receiving the treatment with umifenovir did not show better outcome than the control group in terms of clinical deterioration or viral clearance 34 (**Table 3**). On the other hand, a meta-analysis that included this RCT 34 and four observational studies on COVID-19 demonstrated that umifenovir treatment enhanced the rate of viral clearance on day 14 (RR 1.27, 95% CI 1.04 to 1.55; *P* = 0.02; *I*^2^ = 63%; n = 683) 35 (**Table 4**).

#### Lopinavir/ritonavir

In two human non-RCTs on SARS, the treatment group performed better with respect to the overall mortality rate or the incidence of ARDS 36, 37. An ongoing RCT on MERS involved a combination of lopinavir/ritonavir and IFN-β 19, which has been shown to be effective in two *in vivo* studies on MERS 38, 39 (**Table 1**). In an RCT on COVID-19, treatment with lopinavir/ritonavir was not associated with a mortality rate reduction at day 28 (treatment group 19.2% *vs.* control group 25.0%; difference, -5.8%; 95% CI -17.3% to 5.7%) 40. In the aforementioned RCT on COVID-19 comparing a combination of lopinavir/ritonavir and umifenovir with standard treatment 34, and in another RCT on COVID-19 comparing “lopinavir/ritonavir plus IFN-α with or without ribavirin” with “ribavirin plus IFN-α” 41, treatment with lopinavir/ritonavir did not show superior outcomes in terms of clinical deterioration or viral clearance (**Table 3**). In meta-analyses on COVID-19 involving two of these RCTs 34, 40, treatment with lopinavir/ritonavir was not associated with clinical recovery or viral clearance 22, 42, 43 (**Table 4**).

#### Ribavirin

Ribavirin has been investigated in previous studies on SARS and MERS, but the results were not consistent (**Table 1**). Although one RCT for a combination therapy of ribavirin and lopinavir/ritonavir in SARS was registered 18, it seems unlikely that this trial can be finished, as SARS has not occurred for a while. In two *in vitro* studies on COVID-19, ribavirin did not show therapeutic effects 29, 30 (**Table S1**). In the aforementioned RCT on COVID-19, comparing “ribavirin plus interferon IFN-α with or without lopinavir/ritonavir” with “lopinavir/ritonavir plus IFN-α”, treatment with ribavirin did not show better outcome in terms of the clinical deterioration or viral clearance 41. In another RCT on COVID-19, a combination of ribavirin and IFN-β1b was evaluated: In the treatment group, the time taken to achieve a National Early Warning Score 2 (NEWS2) (4 [IQR 3-8] *vs.* 8 7-9 days; HR 3.92, 95% CI 1.66 to 9.23) or sequential organ failure assessment (SOFA) score of zero (3.0 [IQR 1.0-8.0] *vs.* 8.0 [6.5-9.0] days; HR 1.89, 95% CI 1.03 to 3.49), the hospitalization period (9.0 [IQR 7.0-13.0] *vs.* 14.5 [9.3-16.0] days; HR 2.72, 95% CI 1.2 to 6.13), and time to viral clearance (7 [IQR 5-11] *vs.* 12 8-15 days; HR 4.37, 95% CI 1.86 to 10.24, *P* = 0.001) were shorter than those of the control group 44 (**Table 3**).

#### Other antiviral agents

In a small-scale RCT on mild to moderate COVID-19, azvudine (FNC) treatment showed better outcome with respect to radiological improvement (*P* = 0.0401) and viral clearance (*P* = 0.0011) 45*.* In the other RCTs on COVID-19, triazavirin 46 or a combination of darunavir and cobicistat 47 did not show therapeutic effects (**Table 3**). Nelfinavir showed therapeutic effects in one 48 out of two *in vitro* studies on SARS (**Table 1**) and in an *in vitro* study on COVID-19 49 (**Table 2**).

### 4-Aminoquinoline

In a study on SARS, chloroquine showed an effect *in vitro* but not *in vivo*, and these results were similar for amodiaquine 50. The results of two *in vitro* studies of chloroquine for MERS conflicted with each other 51, 52. Multiple *in vitro* studies on COVID-19 reported effects of chloroquine 29, 53, 54 and hydroxychloroquine 53, 54. In a preprint RCT on COVID-19, hydroxychloroquine was administered with a daily dosage of 400 mg for five consecutive days and the treatment group showed higher rates of improvement in chest computed tomography (CT) scans on day 6 (25 out of 31 [81%] *vs.* 17 out of 31 [55%], *P* = 0.0476) and shorter duration of fever (2.2 [standard deviation (SD) 0.4] *vs.* 3.2 [1.3] days, *P* = 0.0008) 55. In another preprint RCT conducted in Pakistan enrolling 500 patients with mild COVID-19, the proportion of patients with negative viral PCR results within seven days was higher in the hydroxychloroquine-treated group (182 out of 349 [52%] *vs.* 54 out of 151 [36%], *P* = 0.001) 56. However, in the other four RCTs and one preprint RCT on COVID-19 involving a total of 815 participants, treatment with hydroxychloroquine did not show better outcome compared to standard treatment (**Table 3**). In 15 meta-analyses on COVID-19, treatment with hydroxychloroquine showed no therapeutic effect and higher risk for adverse events. A combination of hydroxychloroquine and azithromycin was also evaluated in three meta-analyses on non-RCTs for COVID-19 and showed a harmful effect (**Table 4**).

### Azithromycin

In an RCT on azithromycin for COVID-19, the hospitalization period of the treatment group was shorter than that of the control group (4.6 [SD 2.6] *vs.* 6.0 [SD 3.2] days, *P* = 0.02) 57. However, in another RCT on azithromycin involving 397 patients with severe COVID-19, azithromycin did not show any therapeutic effect 58 (**Table 3**).

### Corticosteroids

In an RCT targeting SARS, early administration (within 7 days) of corticosteroids was associated with higher subsequent plasma viral concentrations in the second and third week of the illness 59. However, in this study, the severity of disease did not differ between the early corticosteroid treatment group and the control group. In addition, there was no significant difference in the median time for the virus to become undetectable in plasma between the early corticosteroid treatment group and the control group (12 *vs.* 8 days, *P =* 0.106). Therefore, it is difficult to conclude that this study supports the risk of corticosteroid treatment. A non-RCT on SARS demonstrated that corticosteroid therapy was associated with higher risk for either ICU admission or mortality (OR 20.7, 95% CI 1.3-338.0) 60. This study had several important limitations, including the following: (1) the 95% CI was extremely asymmetric; (2) there was no difference in mortality between the steroid-treated and non-treated groups in a simple univariate analysis, but corticosteroid therapy was included in the logistic regression; (3) the steroid-treated group had a more severe disease course, which indicates a case of confounding by indication; and (4) not all of the potential variables were adjusted, which could influence the results.

In a non-RCT on MERS, corticosteroid therapy was not associated with 90-day mortality but associated with delay in viral clearance (adjusted HR 0.35, 95% CI 0.17-0.72) under a marginal structural model 61. However, this study also shared many of the shortcomings mentioned above such as corticosteroids being used for patients with severe conditions, which can introduce severe levels of bias. In this retrospective study, at least a propensity-score matching analysis should have been considered.

Three non-RCTs of corticosteroid use in SARS showed effectiveness of high dose 62-64. One non-RCT on SARS demonstrated that the survival outcome of the group receiving methylprednisolone was superior compared to the group not receiving corticosteroids as well as the group receiving hydrocortisone or pulse therapy 65. In an RCT on methylprednisolone treatment for severe COVID-19, the treatment group showed a lower mortality rate (2 out of 34 [6%] *vs.* 12 out of 28 [43%], *P* < 0.001) and a higher rate of clinical improvement (32 out of 34 [94%] *vs.* 16 out of 28 [57%], *P* = 0.001) compared to the control group 66. Meta-analyses on corticosteroid therapy for COVID-19 included only non-RCTs and did not demonstrate any significant therapeutic effect of corticosteroids 67-69 (**Table 4**).

### Colchicine

In two RCTs on COVID-19, colchicine showed effects in major outcomes. Among them, in a Greek RCT, the treatment group had a higher cumulative event-free 10-day survival rate (97% *vs.* 83%, *P* = 0.03), a longer event-free survival period (21 [SD 0.31] *vs.* 19 [0.83] days, *P* = 0.03), and a lower incidence of deterioration within three weeks (2% *vs.* 14%; OR 0.11, 95% CI 0.01 to 0.96; *P* = 0.046) 70. In another preprint RCT conducted in Brazil, the treatment group had a shorter duration of supplemental oxygen therapy (3.0 [IQR 1.5-6.5] *vs.* 7.0 [3.0-8.5] days, *P* = 0.02), a lower proportion of participants requiring supplemental oxygen on day 7 (6% *vs.* 39%, *P* = 0.01), a shorter length of hospital stay (6.0 [IQR 4.0-8.5] *vs.* 8.5 [5.5-11.0] days, *P* = 0.03), and lower rate of hospitalization (53% *vs.* 78% on day 5; 6% *vs.* 17% on day 10; *P* = 0.01) 71.

### ACEI or ARB

In a preprint RCT on 82 participants with COVID-19, telmisartan was administered to the treatment group with a daily dosage of 160 mg for 14 consecutive days. The treatment group had a shorter duration of hospital stay (9 *vs.* 15 days, *P* = 0.0124) and the HR for hospital discharge was 2.02 (95% CI 1.14 to 3.59) 72 (**Table 3**). In a meta-analysis that included three non-RCTs on COVID-19 with hypertension, ARB showed a survival benefit (OR for mortality 0.51, 95% CI 0.29 to 0.90, *P* = 0.02; *I*^2^ = 22%, n = 484) although there was a publication bias 73. In another meta-analysis that included non-RCTs on COVID-19, the effect of ACEI or ARB therapy on COVID-19 with hypertension was not significant 74 (**Table 4**).

### Anticoagulants

In a small-scale RCT for severe COVID-19, therapeutic anticoagulant therapy with enoxaparin and prophylactic anticoagulant therapy with enoxaparin or unfractionated heparin were compared. A greater proportion of participants in the therapeutic anticoagulant group were able to be weaned from MV successfully compared to the prophylactic anticoagulant group (8 out of 10 [80%] *vs*. 3 out of 10 [30%]; HR 4.0, 95% CI 1.04 to 15.05; *P* = 0.031) 75 (**Table 3**). In two meta-analyses including non-RCTs on COVID-19, anticoagulant therapy did not show a therapeutic effect 76, 77 (**Table 4**).

### Calcifediol

Calcifediol was studied in one RCT on COVID-19. In this RCT, a lower proportion of participants in the treatment group were admitted to the ICU compared to the control group (1 out of 50 [2%] *vs.* 13 out of 26 [50%]; adjusted OR 0.03, 95% CI 0.003 to 0.25) 78 (**Table 3**).

### CM4620-IE (Auxora^TM^, calcium release-activated calcium channel inhibitor)

In an RCT on severe or critical COVID-19, the proportion of patients who received invasive MV or died was lower in the Auxora-treated group than the control group (3 out of 17 [18%] *vs.* 5 out of 9 [6%]; HR 0.23, 95% CI 0.05 to 0.96; *P* < 0.05) and the mean difference in the 8-point ordinal scale was statistically significant on day 6 and day 9 to 12 (*P* < 0.05) 79 (**Table 3**).

### Janus-associated kinase inhibitors

In an *in vitro* study on MERS, baricitinib showed an effect 80 (**Table 1**). Ruxolitinib was evaluated in an RCT on severe COVID-19 and showed higher rates of improvement on chest CT scans on day 14 (18 out of 20 [90%] *vs.* 13 out of 21 [62%], *P* = 0.0495) and shorter time to lymphocyte recovery (5 [IQR 2-7] *vs.* 8 2-11 days, *P* = 0.033) 81 (**Table 3**).

### Leflunomide (dihydroorotate dehydrogenase inhibitor)

In a small-sized RCT on moderate COVID-19, the duration of viral shedding was shorter in the leflunomide-treated group compared with the control group (5 *vs.* 11 days, *P* = 0.046) 82 (**Table 3**).

### Immunotherapy

#### Interferon

Both IFN-α and IFN-β showed numerous positive results in non-human studies on SARS or MERS. However, a meta-analysis on MERS did not show that the interferon therapy was effective 14. For COVID-19, five RCTs on interferon have been published to date. In an RCT evaluating IFN-β1a treatment, the rate of hospital discharge was higher (67% *vs.* 44%; OR 2.5, 95% CI 1.05 to 6.37) and the 28-day mortality rate was lower (19% *vs.* 44%, *P* = 0.015) in the treatment group compared to the control group 83. In the aforementioned RCT evaluating a combination of IFN-β1b and ribavirin, the treatment group performed better 44. In another RCT on severe COVID-19, treatment with IFN-β1b showed better outcomes in terms of discharge from the hospital and admission to the ICU compared to standard treatment 84. Inhaled IFN-κ plus TFF2 therapy was investigated in one RCT on moderate COVID-19. In this study, the chest CT findings of participants in the treatment group improved within a shorter time compared to the control group (6.2 [95% CI 5.1-7.3] *vs.* 8.8 [95% CI 7.6-10.0] days, *P* = 0.002) 85. In an RCT evaluating Novaferon therapy, the group receiving the combination of Novaferon and lopinavir/ritonavir had a higher rate of viral clearance on day 6 (18 out of 30 [60%] *vs.* 7 out of 29 [24%], *P* = 0.0053) and a shorter median time to negative results of virus PCR (6 *vs.* 9 days, *P* = 0.036) compared with the group receiving lopinavir/ritonavir 86 (**Table 3**).

#### Convalescent plasma

A meta-analysis of convalescent plasma therapy in patients with SARS demonstrated that the absolute reduction in the risk of mortality was 7% and 23% in two studies, and the case fatality rate from four non-comparative studies varied from 0% to 12.5% 12. In an RCT for severe or life-threatening COVID-19, the proportion of participants who recovered clinically within 28 days was higher in the treatment than in the control group in cases with severe COVID-19 (21 out of 23 [91%] *vs.* 15 out of 22 [28%]; HR 2.15, 95% CI 1.07 to 4.32) 87. Four preprint RCTs on COVID-19 have investigated the effect of convalescent plasma. In one of these RCTs, a lower proportion of participants in the treatment group compared to the control group either required MV or died (0 out of 38 [0%] *vs.* 6 out of 43 [14%], *P* = 0.03) 88. Another RCT on moderate COVID-19 involving more participants (n = 464) did not show the therapeutic effect of convalescent plasma 89 (**Table 3**).

#### Recombinant human granulocyte colony-stimulating factor (rhG-CSF)

In an RCT for COVID-19 with lymphopenia, participants in the treatment group who received rhG-CSF showed lower rates of 21-day mortality (2 out of 100 [2%] *vs.* 10 out of 100 [10%]; HR 0.19, 95% CI 0.04 to 0.88) and disease progression (2 out of 100 [2%] *vs.* 15 out of 100 [15%]; mean difference -13%, 95% CI -21.4% to -5.4%) 90 (**Table 3**).

#### Intravenous immunoglobulin

In a preprint RCT on COVID-19, the 3-day course of intravenous immunoglobulin therapy showed a lower rate of MV within 30 days (2 out of 14 [14%] *vs.* 7 out of 12 [58%], *P* = 0.038), shorter hospital stay (11 [range 5-22] *vs.* 19 4-30 days, *P* = 0.013) or ICU stay (2.5 [range 0-16] *vs.* 12.5 1-29 days, *P* = 0.006), and improvement in PaO_2_/FiO_2_ ratio on day 7 (difference +131 [+35 to +330] *vs.* +44.5 [-115 to +157], *P* = 0.01) among the participants who had an alveolar-arterial oxygen gradient greater than 200 mmHg at enrollment 91 (**Table 3**).

#### Other immunotherapies

In a small-scale RCT on severe COVID-19, vilobelimab (anti-C5a antibody IFX-1) treatment did not show a therapeutic effect 92. In a small-scale RCT on CIGB (anti-CK2) for COVID-19, there was a reduction in the number of pulmonary lesions on chest CT in a greater proportion of participants in the treatment group compared to the control group (5 out of 6 [83%] *vs*.3 out of 7 [43%]; Bayesian *P* (difference > 0) = 0.951) 93. Multiple observational clinical studies on tocilizumab (anti-interleukin [IL] -6 receptor antibody) for COVID-19 were investigated in two meta-analyses. Among them, a subgroup analysis, in which lopinavir and ritonavir were and corticosteroids were not administered to all participants, showed a lower mortality rate in the tocilizmub treatment group (risk difference -0.31, 95% CI -0.57 to -0.05) 94 (**Table 4**).

## Discussion

We summarized the results of studies conducted on SARS, MERS, and COVID-19 to date. Unfortunately, completed RCTs for the treatment of SARS and MERS were scarce. We assumed this was because SARS was a relatively short-lived epidemic that has not occurred since 2004, and the number of patients with MERS might have been insufficient for recruitment. In the case of COVID-19, numerous RCTs have been registered, and research results have been consistently reported despite the global pandemic and medical crisis.

It was difficult to find an optimal therapeutic agent that consistently resulted in positive outcomes across SARS, MERS, and COVID-19. One of the possible reasons of this is that there might not be a universal “cure” to these viral diseases given the differences in presentation forms. Reduction of the viral load may not be the only aim when attempting to cure the disease. The subtle differences between these three coronaviruses, as well as the lack of objective information from clinical experiences of the preceding SARS and MERS epidemics, may also be other reasons.

Synthesizing studies on COVID-19 highlighted two main goals in the treatment of COVID-19: (1) effective elimination of the virus and (2) immune regulation to interfere with the mechanisms of cytokine storm. Therefore, extensive further research on various antiviral agents and immunomodulators is expected to continue for a while.

Among the antiviral agents presented in our study, remdesivir has consistently shown potent effects in non-human studies on SARS, MERS, and COVID-19. Remdesivir is a novel broad-spectrum antiviral agent. When remdesivir is administered into the human body, it is metabolized to an active metabolite, which is an adenosine nucleoside triphosphate analogue. It interferes with the action of viral RNA polymerase and evades proofreading by viral exoribonuclease (ExoN), which interferes with RNA replication of the virus 95. This agent showed an effect in the largest of the four RCTs on COVID-19 that included 1,062 participants. In this RCT, the 14-day mortality rate was significantly lower in the subgroup with requirement of only supplemental oxygen not MV, suggesting that there is a patient population with a certain level of severity of COVID-19 that could benefit from remdesivir treatment 20. Therefore, it seems necessary to preferentially administer remdesivir to this specific group of patients. In addition, it should be considered in the clinical application that the outcome of 5-day regimen treatment was better than that of the 10-day regimen in two RCTs 23, 24. On October 22, 2020, the Food and Drug Administration (FDA) approved the use of remdesivir for treatment of COVID-19 requiring hospitalization.

A combination of sofosbuvir and daclatasvir consistently showed an effect on the major outcomes in two RCTs on COVID-19. Sofosbuvir is a nucleotide analog targeting the hepatitis C virus (HCV) polymerase, NS5B. This agent is capable of inhibition of positive-strand RNA viruses like coronavirus. Daclatasvir is an HCV NS5A antagonist and is known to penetrate lung tissue effectively. Daclatasvir has been shown to inhibit the production of SARS-CoV-2 particles in an *in vitro* study 96. Those two RCTs investigating the combination of sofosbuvir and daclatasvir had limitations because they included a small number of participants and did not show significant results for mortality. Therefore, a large-scale RCT on this treatment is still required.

Favipiravir is an antiviral agent that is drawing attention as a treatment option for COVID-19. The active metabolite of favipiravir competes with purine nucleosides and incorporates into viral RNA to interfere with viral replication, potentially inhibiting the RNA dependent RNA polymerase (RdRp) of RNA viruses 97. Considering that favipiravir showed an effect in terms of viral clearance in an RCT on COVID-19 31, a large-scale, well-designed RCT is further required. A number of RCTs on favipiravir are now underway.

Umifenovir is a small indole-derivative molecule with broad-spectrum antiviral property, and it has been approved in Russia and China for the prophylaxis and treatment of influenza. It inhibits the virus from fusion to the target cell membrane and blocks viral entry into the cell 98. Since a meta-analysis including one RCT and multiple observational studies on umifenovir for COVID-19 demonstrated significant results for viral clearance 35, well-designed RCTs on umifenovir still needs to be conducted.

Lopinavir/ritonavir combination therapy seems to have more therapeutic effect than monotherapy of each drug. As protease inhibitors, lopinavir and ritonavir can inhibit the action of 3CLpro, coronavirus main protease, and interfere with the process of viral replication and release 99. However, the clinical results of this combination therapy for COVID-19 were not meeting expectations compared to those of SARS and MERS. In a first RCT on lopinavir/ritonavir for COVID-19, the outcomes of the treatment group and the control group did not show a significant difference, but the 28-day mortality of the treatment group were slightly lower 40; and in two other RCTs and four meta-analyses on COVID-19, treatment with lopinavir/ritonavir did not show an effect.

Azvudine (FNC) is a novel nucleoside reverse transcriptase inhibitor targeting HCV and has been investigated for treatment of human immunodeficiency virus (HIV) 100. Azvudine showed promising effects in terms of radiological improvement and viral clearance in a small-scale RCT for mild to moderate COVID-19 45. RCTs involving more participants are required.

The 4-aminoquinoline is mainly used as an anti-malaria agent, and hydroxychloroquine, one of its derivatives, is also used as an immunomodulatory agent. Chloroquine and hydroxychloroquine inhibits the pH-dependent steps of viral replication by increasing the pH of phagolysosome 101. The study on SARS showed *in vitro* but not *in vivo* effects of chloroquine and amodiaquine 50, and combination therapy with other drugs that inhibit viral replication may be necessary. In our study, we investigated seven RCTs and 15 meta-analyses on hydroxychloroquine for COVID-19 and only one preprint small-scale RCT demonstrated an effect of hydroxychloroquine on the major outcomes 55. The survival benefit of hydroxychloroquine treatment has not been demonstrated in any of these seven RCTs. The results of the latest large-scale RCT 102, which was excluded from our study since it included patients with suspected COVID-19, as well as multiple meta-analyses were consistent with this. In addition, the risk for adverse events of hydroxychloroquine treatment was higher compared to standard treatment according to the results of meta-analyses. Therefore, hydroxychloroquine seems to have no value as a therapeutic agent for COVID-19.

It is worth noting that an RCT for chloroquine, excluded due to involvement of patients with suspected COVID-19 103, suggested the risk of high-dose chloroquine treatment and this risk was associated with prolongation of the QT interval. Similarly, hydroxychloroquine treatment was also related to QT prolongation 104, 105. Therefore, it is expected that if careful monitoring for prolongation of the QT interval is not accompanied, chloroquine or hydroxychloroquine treatment can be rather harmful. In this regard, the World Health Organization (WHO) recently decided to implement the temporary pause of the hydroxychloroquine arm within the Solidarity Trial, a large-scale study on four untested treatments for COVID-19 106. The FDA also withdrew emergency use authorization for chloroquine and hydroxychloroquine 107.

For severe inflammatory diseases caused by infection, corticosteroid therapy is a double-edged sword. Although a number of corticosteroid therapies have already been used in SARS, MERS, and COVID-19, these results are controversial and difficult to interpret. It is noteworthy that the study of SARS showed a difference in outcomes depending on the type or dosage of steroids 65. In most observational studies on COVID-19, corticosteroid therapy was mainly administered in a group with severe clinical conditions according to the prevailing guidelines 7. Because of this strong tendency, patients with COVID-19 who received corticosteroids had poor treatment outcomes, and objective validation of corticosteroid treatment has been highly required.

In response to this request, the results of the first RCT to investigate corticosteroid therapy in COVID-19 were recently reported, although it was excluded from our study because participants with negative SARS-CoV-2 PCR results were included. This RCT involved 2,104 and 4,321 participants in the treatment and control group, respectively. The 28-day mortality rate of the group receiving 10 days of dexamethasone treatment was lower than that of the control group (482 out of 2,104 [23%] *vs.* 1,110 out of 4,321 [26%]; RR 0.83, 95% CI 0.75 to 0.93; *P* < 0.001), and this trend was stronger among the subgroup with higher severity of COVID-19: (1) 95 out of 324 patients (29%) in the treatment group and 283 out of 683 patients (41%) in the control group among patients requiring invasive MV (RR 0.64, 95% CI 0.51 to 0.81) (2) 298 out of 1,279 patients (23%) in the treatment group and 68 out of 2,604 patients (26%) in the control group among patients requiring only oxygen supplement (RR 0.82, 95% CI 0.72 to 0.94). There was no significant difference in the 28-day mortality rate among patients not receiving respiratory support 108. These results strongly suggest that dexamethasone treatment is effective for a population of patients with COVID-19 requiring respiratory support. It is necessary to confirm these results among the participants who were diagnosed as COVID-19 through viral RNA detection. In another RCT for severe COVID-19, patients receiving methylprednisolone also showed a lower mortality rate compared to the control group 66.

Colchicine, an anti-inflammatory agent which is mainly used for gout and rheumatoid arthritis, has been used for a long time. It inhibits microtubule polymerization and mitosis in the metaphase 109. It is promising that the effects of colchicine treatment were revealed in terms of survival, clinical improvement, and duration of hospitalization in the two RCTs for COVID-19 70, 71. If a larger-scale follow-up RCT is conducted, the effect might be further supported.

Angiotensin-converting enzyme 2 (ACE2) is a transmembrane protein and the main entry point into cells for SARS-CoV-2. Theoretically, if the expression of ACE2 decreases, it will be a defense mechanism against the entry of the virus. On the other hand, ACE2 shows a protective action against virus-induce lung injury by converting angiotensin II to angiotensin-(1-7), which have a vasodilator effect 110, 111. ACEI and ARB can induce up-regulation of ACE2 112, 113, which might negatively affect the treatment of disease. Contrary, an *in vivo* study showed that SARS-CoV spike-mediated lung injury was attenuated by losartan 114. For these conflicting evidences, there has been an interest in ACEI and ARB in relation to COVID-19. In one RCT for COVID-19 72 and one meta-analysis including three non-RCTs on COVID-19 73, participants who received ARB performed better in terms of discharge or survival. More RCTs on ACEI or ARB for COVID-19 in the group of patients with pre-existing hypertension or at risk for cardiovascular disease are still required.

Routine administration of anticoagulants in sepsis or ARDS is not recommended currently. However, disseminated intravascular coagulation should still be the target of research to find treatments for sepsis or ARDS, because it is deeply involved in the pathogenesis and progress of these diseases. It has been reported that coagulopathy was associated with the prognosis of COVID-19 115-117 and these results are consistent with what has been known in ARDS and sepsis. In a small-scale RCT of severe COVID-19, therapeutic anticoagulant therapy with enoxaparin showed a better outcome than prophylactic anticoagulant therapy 75. More studies investigating the efficacy of augmenting these anticoagulation or thrombolytic treatments, while weighing the risk of hemorrhage, and narrowing the indications are required.

Calcifediol is a main metabolite of vitamin D. Since lung epithelium expresses vitamin D receptors, administration of vitamin D may suppress the development of ARDS 118. In a pilot RCT on COVID-19, treatment with a high dosage of calcifediol reduced the need for ICU admission 78.

CM4620-IE (Auxora^TM^) is a selective small molecule inhibitor of calcium release-activated calcium (CRAC) channels. It was developed to prevent over-activation of CRAC channels that can lead to inflammatory diseases. It has been suggested that Auxora may protect against pulmonary endothelial damage and cytokine storm 119, 120. This agent has been shown to be effective in terms of survival and clinical improvement in one small-scale RCT for severe or critical COVID-19 79.

Ruxolitinib is a potent selective inhibitor of Janus-associated kinases 1 and 2 and has been used as a treatment for primary myelofibrosis, post-polycythemia vera or post-essential thrombocythemia myelofibrosis 121. Ruxolitinib has a broad-spectrum of anti-inflammatory properties against cytokine storm mediated by IL-1, IL-6, IL-8, IL-12, tumor necrosis factor-α, IFN-γ, vascular endothelial growth factor, and granulocyte-macrophage colony-stimulating factor 122. In an RCT on severe COVID-19, ruxolitinib showed higher rate of radiological improvement despite the small number of participants 81. In this RCT, no one out of 20 patients with severe COVID-19 in the treatment group died within 28 days, whereas three out of 21 patients in the control group died. Further research results for severe COVID-19 at high risk for cytokine storm need to be supplemented.

Leflunomide, an isoxazole derivative, inhibits the T cell proliferation by blocking dihydroorotate dehydrogenase. This agent has been used in the treatment of rheumatoid arthritis and psoriatic arthritis, and has been attempted to treat BK virus, cytomegalovirus, HIV, and ebolavirus 123. The only RCT evaluating leflunomide for COVID-19 enrolled only 10 participants, but showed an effect of shortening the viral shedding period 82. Similarly, in a preprint observational study involving 27 participants, leflunomide showed effects of promoting viral clearance and increasing discharge rate 124.

Interferon showed effects in a number of *in vitro* studies on SARS and MERS. In addition, three RCTs on IFN-β for COVID-19 demonstrated favorable results in terms of survival, clinical improvement, discharge from hospital, and viral clearance 44, 83, 84. Since interferon has been used as a combination therapy with antiviral agents in most cases, further research is needed to discover the antiviral agent that can show the greatest effect when administered in combination with interferon, as well as specific indications.

Convalescent plasma contains pathogen-specific neutralizing antibodies that can neutralize viral particles, which provide passive immunity to the recipient. It is hypothesized that early convalescent plasma therapy enhances the patient's capability to clear the initial viral inoculum by neutralizing viral particles 125. Convalescent plasma therapy has been applied to a wide range of infectious diseases such as diphtheria, pneumococcal pneumonia, hepatitis A and B, mumps, polio, measles, and rabies. The results of a meta-analysis on convalescent plasma treatment for SARS are relatively promising 12. In one RCT for severe COVID-19, convalescent plasma treatment showed an effect in terms of clinical improvement 87. In contrast, the effect of convalescent plasma treatment was not demonstrated in another RCT on moderate COVID-19 involving larger number of participants 89. The differences in severity of COVID-19 in these two RCTs may have contributed to this contradiction. Therefore, a large-scale RCT on convalescent plasma treatment targeting severe COVID-19 is required.

Intravenous immunoglobulin therapy provides passive immunity and has the property to modulate immune function. High doses of intravenous immunoglobulin can produce anti-inflammatory and inflammatory-modulating effects on a variety of immune cells, which can intervene and modulate the mechanisms of cytokine storm, and have been administered to treat various diseases such as immune thrombocytopenia purpura or Kawasaki disease 126. The only preprint small-scale RCT on intravenous immunoglobulin therapy for COVID-19 showed better clinical outcomes in the treatment group 91.

Our study has some limitations. RCTs for SARS and MERS were extremely rare. In the case of COVID-19, more RCTs were obtained. However, all except for eight RCTs included less than 100 participants per each arm. Although we updated the latest search results for RCTs and meta-analyses on COVID-19, we did not include the latest search results for non-clinical studies on the three coronavirus diseases because of the vast amount of data.

In addition, our study also highlights that treatments with potential effects seen in *in vitro* studies have not translated in positive *in vivo* or clinical studies. The 4-aminoquinoline derivatives showed effects in a number of *in vitro* studies, but not in *in vivo* and clinical studies. On the other hand, favipiravir showed unfavorable results in an *in vitro* study, while it showed effects in a clinical study. This contradiction between *in vitro* and *in vivo* studies or between pre-clinical and clinical studies does not help in the current situation where a therapeutic agent for COVID-19 must be discovered in a short amount of time. Therefore, it is important to design *in vivo* or clinical studies after a thorough understanding of drug pharmacology and in-depth consideration of how to link *in vitro* antiviral activity and drug exposure at the putative target site of action. Fan *et al.* demonstrated that *in vitro* EC_50_/EC_90_ values for hydroxychloroquine should be compared to the *in vivo* free extracellular tissue concentration, which is similar to the free plasma hydroxychloroquine concentration 127. Advances in cell modeling tools for biological research are expected to further enrich preclinical research design, and also help promote the development of new therapies 128.

When a specific infection enters a pandemic state, group immunization through vaccine, rather than quarantine, is the most effective countermeasure. As of October 29, 2020, 201 candidate vaccines against SARS-CoV-2 are being developed. Among them, 45 have entered clinical trials, and none has been approved for use yet 129.

## Conclusion

In this summary report, we synthesized the results of previous studies on the treatment of SARS, MERS, and COVID-19. There was no therapeutic agent that consistently resulted in positive outcomes across SARS, MERS, and COVID-19. Remdesivir showed a therapeutic effect for COVID-19 in two RCTs involving the largest number of total participants (n = 1,461). Other therapies that showed an effect in at least two RCTs for COVID-19 were sofosbuvir/daclatasvir (n = 114), colchicine (n = 140), IFN-β1b (n = 193), and convalescent plasma therapy (n = 126). Further RCTs are required.

## Supplementary Material

Supplementary table S1.

## Figures and Tables

**Table 1:** Summary of studies evaluating therapeutics for SARS and MERS

Therapeutics	SARS			MERS		
	*In vitro*	*In vivo*	Human	*In vitro*	*In vivo*	Human
Antiviral agents						
Ribavirin	4 studies 36, 130-132 **2 studies 133, 134**		4 studies 135-138	1 study 139		
Remdesivir	1 study 140	1 study 140	4 studies 38, 140-142	2 studies 38, 143		
Lopinavir	1 study 130 **2 studies 48, 50**			2 studies 38, 51 **1 study 95**		
Ritonavir	1 study 48					
Oseltamivir			**1 study 136**			**1 study 144**
Nelfinavir	1 study 48 **1 study 50**	**1 study 50**				
**Interferon**	IFN-α (8 studies) 50, 130, 132, 134, 145-148; IFN-β (8 studies) 130, 132, 145, 147-151	IFN-α/IL-1β (1 study) 152 IFN-α B/D, rintatolimod† (1 study) 50.	IFN-α (1 study): more effective than corticosteroids 153.	IFN-α (1 study) 139; IFN-β (2 studies) 154, 155.	IFN-β (2 studies) 39, 156§	**1 study 14‡**
		**IFN-α-n3 (1 study) 50**				
**Combination therapy based on antiviral agents or interferon**						
Ribavirin/IFN	IFN-α (1 study) 130; IFN-β (2 studies) 130, 131.			IFN-α (1 study) 157		IFN-α (2 studies) CFR 6/20 (30%) *vs.* 17/24 (71%) (*P =* 0.01) 158 CFR 14/61 (23%) *vs.* 2/2 (100%) (*P =* 0.01) 159.
						**4 studies 144, 160-162: no difference in mortality.**
Ribavirin/lopinavir	1 study 36					
Ribavirin plus L/r			Registered RCT(not yet recruiting) 18			
L/r	3 studies 36, 99, 130 **1 study 48**		2 studies: Rates of ARDS/death (2% *vs.* 29%, *P =* 0.001) 36 CFR 2% *vs.* 16% (*P <* 0.05) 37.	1 study 38	1 study 39	
L/r plus IFN-β				2 studies 38, 39	Ongoing RCT 19	
Ribavirin/corticosteroids		1 study earlier administration 163				
IFN-α/corticosteroids		1 study 153				
IFN-β/IFN-γ	1 study 164					
Intranasal IFN-β/ HR2P-M2				1 study 156		
***Antibiotics***						
**Macrolide**					**1 study: mortality and viral clearance 165**	
**4-Aminoquinoline**						
Chloroquine	3 studies 50, 166, 167	**1 study 50**		1 study 51 **1 study 52**		
Amodiaquine	1 study 50	**1 study 50**				
**Corticosteroids**			4 studies Higher dose 62, 63 High dose methylprednisolone 64 Methylprednisolone was better than 3 other groups (no steroid, hydrocortisone, or pulse therapy) 65.		Inconclusive (2 studies); Delay in viral clearance (HR 0.35; 95% CI 0.17-0.72), not associated with mortality 61 CFR 6/13 (46%) *vs.* 2/19 (11%) (*P =* 0.04) in univariate analysis 144.	
			Inconclusive (2 studies) 59, 60 Early administration - higher plasma viral load, no difference in severity 59* Possible adverse effect (1 study): osteonecrosis 10‡.			
**Immunotherapy**						
Convalescent plasma			Inconclusive (1 study) CFR [0/1 (0%) *vs.* 2/28 (7%)] and [0/19 (0%) *vs.* 5/21 (24%) in 2 comparative studies, and 0/1, 0/1, 0/3, and 10/80 in 4 non-camparative studies 12‡	1 study 168	Inconclusive (1 study): 2 out of 3 cases with respiratory failure showed neutralizing activity 169.	
Monoclonal antibody		201 170		m336 168, hMS-1 171, 4C2h 172, HR2P-M2 156.		
**Other drugs**	β-D-N4-hydroxycytidine 50; calpain inhibitor VI 50	Camostat 173		Camostat 80, loperamide 51, chlorpromazine 51, 52, 174, imatinib 174, 175, saracatinib 80, 175, baricitinib 80, dasatinib 174, cyclosporine 176, EST 80, cathepsin L/K inhibitor 80, gemcitabine/toremifene/ triflupromazine 174, mycophenolic acid 155.		
		**β-D-N4-hydroxycytidine 50; calpain inhibitor VI 50**		**Toremifene 52**		

(Effective; bold Not effective). In the outcome description, the former is the data of the treatment group and the latter is the data of the control group. CFR: case-fatality ratio; CI: confidence interval; EST: (23,25)-*trans*-epoxysuccinyl-l-leucylamindo-3-methylbutane ethyl ester; HR: hazard ratio; ICU: intensive care unit; IFN: interferon; L/r: lopinavir/ritonavir; MERS: Middle East respiratory syndrome; OR: odds ratio; RCT: randomized controlled trial; RR: risk ratio; SARS: severe acute respiratory syndrome; *This study is the only published randomized controlled trial in this table. †A mismatched double-stranded RNA interferon inducer. ‡Meta-analysis. §Intranasal administration.

**Table 2:** Therapeutic agents that showed effects against SARS-CoV-2 in *in vitro* studies

Therapeutics	First author	Findings	Conclusion
**Antiviral agents**			
Umifenovir	Wang 33	EC_50_ = 4.11 μM; CC_50_ = 31.79 μM; SI = 7.73	Potent
Remdesivir	Wang 29	EC_50_ = 0.77 μM; CC_50_ > 100 μM; SI > 129.87	Potent
	Choy 30	EC_50_ = 26.9 μM; CC_50_ > 100 μM	Not potent
Nelfinavir	Musarrat 49	Complete inhibition of SARS CoV-2 mediated cell fusion at 10 μM	Potent
**Antiparasitic agents**			
Ivermectin	Caly 177	5000-fold reduction in viral RNA at 48h after a single administration (IC_50_ < 2mM)	Potent
Emetine	Choy 30	EC_50_ = 0.5 μM; CC_50_ = 56.46 μM	Potent
**4-aminoquinoline (anti-malarial agents)**			
Chloroquine	Wang 29	EC_50_ = 1.13 μM; CC_50_ > 100 μM; SI > 88.5	Potent
	Yao 53	Incubation time may influence antiviral activity (24h EC_50_ = 23.9 μM; 48h EC_50_ = 5.47 μM).	Potent
	Liu 54	EC_50_ = 2.71 (MOI = 0.01), 3.81 (0.02), 7.14 (0.2), 7.36 (0.8) μM; CC_50_ = 273.2 μM	Potent
Hydroxychloroquine	Yao 53	24h EC_50_ = 6.14 μM; 48h EC_50_ = 0.72 μM	Potent
	Liu 54	EC_50_ = 4.51 (MOI = 0.01), 4.06 (0.02), 17.31 (0.2), 12.96 (0.8) μM; CC_50_ = 249.5 μM	Potent
**Other agents**			
Homoharrngtonine	Choy 30	EC_50_ = 2.14 μM; CC_50_ = 59.75	Potent
Nitazoxanide	Wang 29	EC_50_ = 2.12 μM; CC_50_ > 35.53 μM; SI > 16.76	Potent
**Immunotherapy**			
EK1C4	Xia 178	IC_50_ = 36.5 nM; CC_50_ > 5 μM; SI > 136	Potent

CC_50_: 50% cytotoxic concentration; COVID-19: coronavirus disease 2019; EC_50_: 50% maximal effective concentration; MOI: multiplicity of infection; SARS-CoV-2: severe acute respiratory syndrome coronavirus 2; SI: selectivity index.

**Table 3:** Summary of RCTs evaluating therapeutics for COVID-19

Therapeutics (daily dosage mg) [Common treatment applied to all participants]	First author	Condition	Region	Period of enrollment	No. of participants (treatment - control group)	Outcome of patients or findings: treatment group *vs.* control group (number of participants or the median value [IQR])	Conclusion
***Antiviral agents***							
**Remdesivir**							
200 mg (day 1); 100 mg (day 2-10) *vs.* standard treatment	Beigel 20	Not specified	World-wide*	Feb 21-Apr 19	541 - 521	Improvement in the ordinal score on day 15: OR 1.50, 95% CI 1.18 to 1.91 (*P* = 0.001) 14-day mortality: 32 (6%) *vs.* 54 (10%) (HR 0.70, 95% CI 0.47 to 1.04) 14-day mortality in patients with a baseline ordinal score of 5 (requiring oxygen): 4/222 (2%) *vs.* 19/199 (10%) (HR 0.22, 95% CI 0.08 to 0.58) Time to recovery: 11 (95% CI 9-12) *vs.* 15 (13-19) days (RR for recovery 1.32, 95% CI 1.12 to 1.55; *P* < 0.0001).	Effective
A: 200 mg (day 1); 100 mg (day 2-10) B: 200 mg (day 1); 100 mg (day 2-5). C: Standard treatment.	Spinner 23	Moderate	The US, Europe, Asia	Mar 15-Apr 18	197 (A) 199 (B) 200 (C)	Better clinical status distribution on the 7-category ordinal scale on day 11: OR (B *vs.* C) 1.65 (95% CI 1.09 to 2.48) 28-day mortality: 3 (A, 2%); 2 (B, 1%); 4 (C, 2%).	Effective (5-day treatment)
200 mg (day 1); 100 (day 2-10) *vs.* standard treatment	Wang 21	Severe	China	Feb 6-Mar 12	158 - 78	28-day mortality: 22 (14%) *vs.* 10 (13%) Time to clinical improvement (a 2-point reduction on a 6-category ordinal scale, or discharge from hospital): 21 13-28 *vs.* 23 15-28 days (HR 1.23, 95% CI 0.87 to 1.75)	Not effective
A: 200 mg (day 1); 100 (day 2-5) B: 200 mg (day1); 100 (day 2-10)	Goldman 24	Severe	World-wide†	Mar 6-Mar 26	200 (A) 197 (B)	Clinical improvement of 2 points or more on a 7-category ordinal scale within 14 days: 129 (A, 64%) *vs.* 107 (B, 54%) (difference -6.5%, 95% CI -15.7% to 2.8%) 14-day mortality among patients receiving MV or ECMO: 10/25 (A, 40%) *vs.* 7/41 (B, 17%) Serious adverse event: 42 (A, 21%) *vs.* 68 (B, 35%) (difference 10.8%, 95% CI 2.4% to 19.2%).	Favors 5-day treatment
**Sofosbuvir/daclatasvir**							
400 mg/60 mg for 14 days *vs.* standard treatment [hydroxychloroquine with or without lopinavir /ritonavir]	Sadeghi 27	Moderate/severe	Iran	Mar 26-Apr 26	33 - 33	Duration of hospitalization: 6 4-8 *vs.* 8 days 5-13 (*P* = 0.029) The cumulative incidence of hospital discharge was higher in the treatment group (*P* = 0.041). Clinical recovery within 14 days: 29 (88%) *vs.* 22 (67%) (*P* = 0.076).	Effective
400 mg/60 mg plus ribavirin (1,200) *vs.* hydroxychloroquine and lopinavir/ritonavir with/without ribavirin	Abbaspour Kasgari 28	Moderate	Iran	Mar 20-Apr 8	24 - 24	ICU admission: 0 (0%) *vs.* 4 (17%) (*P* = 0.109) Hospital mortality 0 (0%) *vs.* 3 (13%) (*P* = 0.234). The cumulative incidence of recovery was higher in the treatment group (*P* = 0.033).	Effective
**Favipiravir**							
A: 3,200 mg (day 1); 1,200 (day 2-14); B: 3,600 day (day 1); 1,600 (day 2-14); C: Standard treatment.	Ivashchenko 31	Moderate	Russia	Apr-May	20 (A) 20 (B) 20 (C)	Discharge or achievement of score 2 on WHO-OSCI by day 15: 13 (A, 65%), 17 (B, 85%), and 17 (C, 85%) Viral clearance on day 5: 25/40 (A and B, 63%) *vs.* 6/20 (C, 30%) (*P* = 0.018)	Possible effect
3,600 mg (day 1); 1,600 mg (day 2-10) *vs.* 3,600 mg (day 6); 1,600 mg (day 7-15)	Doi 32	Mild	Japan	Mar 2-May18	36 - 33	Time to discharge from the hospital: 14.0 *vs.*21.5 days (HR 2.68, 95% CI 1.67 to 4.29) Viral clearance on day 6: 67% *vs.* 56% (adjusted HR 1.42, 95% CI 0.76 to 2.62) 69 out of 82 participants (84%) developed hyperuricemia.	Favors early treatment
**Other antiviral agents**							
Lopinavir (800)/ ritonavir (200) for 14 days *vs.* standard treatment	Cao 40	Severe	China	Jan 18-Feb 3	99 - 100	28-day mortality: 19 (19%) *vs.* 25 (25%) (difference -5.8%; 95% CI -17.3% to 5.7%) Time to clinical improvement (a 2-point reduction on a 7-category ordinal scale or discharge from hospital): 16 13-17 *vs.* 16 15-18 days (HR 1.24, 95% CI 0.90 to 1.72) Hospital stay: 14 12-17 *vs.* 16 13-18 days (difference 1, 95% CI 0 to 2)	Not effective
A: Lopinavir (400) /ritonavir (100) for 7-14 days B: Umifenovir (600) for 7-14 days C: Standard treatment	Li 34	Mild/ moderate	China	Feb 1-Mar 28	34 (A) 35 (B) 17 (C)	Deterioration to severe/critical COVID-19 on day 7: 8/34 (A, 24%), 3/35 (B, 9%), and 2/17 (C, 12%) (*P* = 0.206) Time to viral clearance: 9.0 (A; SD 5.0), 9.1 (B; 4.4), and 9.3 (C; 5.2) days (*P* = 0.981) Viral clearance within 7 days: 12/34 (A, 35%), 13/35 (B, 37%), and 7/17 (C, 41% ) (*P* = 0.966).	Not effective
A: Ribavirin (2,000 mg loading; 1,200-1,800 mg for 14 days) B: Lopinavir (800)/ritonavir (200) C: Ribavirin plus lopinavir/ritonavir	Huang 41	Mild/ moderate	China	Jan 29-Feb 25	33 (A) 36 (B) 32 (C)	Deterioration to severe COVID-19: 1 (A, 3%), 2 (B, 6%), and 2 (C, 6%) (*P* = 0.58) Time to viral clearance: 13.0 [9.0-25.5] (A), 12.0 [7.0-19.0] (B), and 15.0 [9.3-17.8] (C) days (*P* = 0.42) Viral clearance on day 14: 17/33 (A, 52%), 22/36 (B, 61%), and 15/32 (C, 47%).	Not effective
Azvudine (FNC) (5) *vs.* standard treatment	Ren 45	Mild/ moderate	China	Feb 18-Feb 29	10 - 10	Time to radiological improvement was shorter in the treatment group (*P* = 0.0401). Viral clearance on day 6: 10 (100%) *vs.* 4 (40%) (*P* = 0.0011).	Effective
Triazavirin (750 or 1,000 for 7 days)‡ *vs.* standard treatment	Wu 46	Not specified	China	Feb 14-Mar 6	26 - 26	Clinical improvement§: 10 (39%) *vs.* 6 (23%) (RR 2.1, 95% CI 0.6 to 7.0, *P* = 0.2) Time to clinical improvement§: 7 6-15 *vs.* 12 7-16 days (RR 2.0, 95% CI 0.7 to 5.6, *P* = 0.2).	Not effective
Darunavir (800)/cobicistat (150) for 5 days *vs.* standard treatment	Chen 47	Mild	China	Jan 30-Feb 6	15 - 15	Worsening of chest CT findings: 7 (47%) *vs.* 4 (27%) (*P* = 0.45) Viral clearance on day 7: 7 (47%) *vs.* 9 (60%) (*P* = 0.72).	Not effective
**Hydroxychloroquine**							
800 mg (day 1); 400 mg (day 2-7) *vs.* standard treatment	Mitjà 179	Mild	Spain	Mar 17-Apr 28	136 - 157	Number of hospitalized participants: 8 (6%) *vs.* 11 (7%) (RR 0.75, 95% CI 0.32 to 1.77); Time to the resolution of symptoms : 10 4-18 *vs.* 12 6-21 days (*P* = 0.38); Reduction in viral load on day 7: -3.49 (SD 0.20) *vs.* -3.37 (0.19) log10 copies/mL (difference -0.12, 95% CI -0.25 to 0.5).	Not effective
1,200 mg (day 1-3); 800 mg (day 4-14) *vs.* standard treatment	Tang 180	Mild/ moderate	China	Feb 11-Feb 29	75 - 75	Alleviation of symptoms by day 28¶: 60% *vs.* 67% (difference -7%, 95% CI -41% to 28%) Viral clearance by day 28: 56 (75%) *vs.* 53 (71%) Adverse events**: 21/70 (30%) *vs.* 7/80 (9%).	Not effective
800 mg (day 1); 400mg (day 2-15) *vs.* standard treatment	Abd-Elsalamb 181	Not specified	Egypt	Mar-Jun	97 - 97	Initiation of MV: 4 (4%) *vs*. 5 (5%) (*P* = 0.75) 28-day mortality: 6 (6%) *vs.* 5 (5%) (*P* = 0.77).	Not effective
1,400 mg (day 1); 600 mg (day 2-5) *vs.* standard treatment	Skipper 182	Mild	The US, Canada	Mar 22-May 20	73 - 72	Change in symptom severity score over 14 days: -2.21(SE 0.23) *vs.* -2.10 (0.23) (*P* = 0.51).	Not effective
800 mg (day 1); 400 mg (day 2-5) *vs.* standard treatment	Kamran 56††	Mild	Pakistan	Apr 10-May 31	349 - 151	Disease progression‡‡: 11 (3%) *vs.* 5 (3%) (*P* = 0.865) Viral clearance within 7 days: 182 (52%) *vs.* 54 (36%) (*P* = 0.001).	Possible effect
400 mg for 5 days *vs.* standard treatment	Chen 55††	Mild/ moderate	China	Feb 4-Feb 28	31 - 31	Improvement of chest CT scans on day 6: 25 (81%) *vs.* 17 (55%) (*P* = 0.0476) Duration of fever: 2.2 (SD 0.4) *vs.* 3.2 (1.3) days (*P* = 0.0008).	Effective
800 mg (day 1); 400 mg (day 2-7) *vs.* standard treatment	Chen 183††	Mild/ moderate	Taiwan	Apr 1-May 31	21 - 12	Clinical recovery (3 consecutive negative results of viral PCR and resolution of major symptoms) within 14 days: 6/21 (29%) *vs.* 5/12 (42%) (*P* = 0.51) Time to viral clearance: 5 (95% CI 1 to 9) *vs.* 10 (2 to 12) days (*P* = 0.40) Viral clearance within 14 days: 17/21 (81%) *vs.* 9/12 (75%) (*P* = 0.36).	Not effective
**Azithromycin**							
500 mg for 10 days *vs.* standard treatment	Furtado 58	Severe	Brazil	Mar 28-May 19	214 - 183	Worse clinical status on the 6-category ordinal scale on day 15: OR 1.36 (95% CI 0.94 to 1.97, *P* = 0.11) 28-day mortality: 90 (42%) *vs.* 73 (40%) (HR 1.08, 95% CI 0.79 to 1.47, *P* = 0.63).	Not effective
500 mg for 5 days *vs.* standard treatment [Lopinavir/ritonavir and hydroxychloroquine]	Sekhavati 57	Not specified	Iran	Apr 24-May 8	56 - 55	Length of hospital stay: 4.6 (SD 2.6) *vs.* 6.0 (SD 3.2) days (*P* = 0.02) Mortality: 0 (0%) *vs.* 1 (2%) (*P* = 0.495) ICU admission: 2 (4%) *vs.* 7 (13%) (*P* = 0.070).	Effective
**Colchicine**							
2 mg (day 1)§§; 1 mg (till discharge or day 21) *vs.* standard treatment [Chloroquine or hydroxychloroquine and azithromycin]¶¶	Deftereos 70	Not specified	Greece	Apr 3-Apr 27	55 - 50	Cumulative event-free 10-day survival rate: 97% *vs.* 83% (*P* = 0.03) Deterioration by 2 points on a 7-category ordinal scale within 3 weeks: 1 (2%) *vs.* 7 (14%) (OR 0.11, 95% CI 0.01 to 0.96, *P* = 0.046) Peak D-dimer concentration: 0.76 [0.41-1.59] *vs.* 0.92 [0.68-2.77] μg/mL (*P* = 0.04).	Effective
1.5 mg (day 1-5); 1 mg (day 6-10) *vs.* standard treatment [azithromycin, hydroxychloroquine, and unfractionated heparin].	Lopes 71††	Moderate/severe	Brazil	Apr 11-Jul 06	17 - 18	Proportion of participants requiring supplemental oxygen on day 7: 6% *vs.* 39% (*P* = 0.01) Maintenance of hospitalization: 53% *vs.* 78% (on day 5), 6% *vs.* 17% (on day 10) (*P* = 0.01) Duration of oxygen supplement: 3.0 [1.5-6.5] *vs.* 7.0 [3.0-8.5] days (*P* = 0.02) Length of hospital stay: 6.0 [4.0-8.5] *vs.* 8.5 [5.5-11.0] days (*P* = 0.03).	Effective
**Other agents**							
Methylprednisolone (250 for 3 days) *vs.*standard treatment [Hydroxychloroquine, lopinavir, naproxen].	Edalatifard 66	Severe	Iran	Apr 20-Jun 20	34 - 28	Mortality: 2 (6%) *vs.* 12 (43%) (*P* < 0.001) Clinical improvement***: 32 (94%) *vs.* 16 (57%) (*P* = 0.001) Time to clinical improvement***: 11.8 (SD 4.9) *vs.* 16.4 (SD 6.9) (*P* = 0.003).	Effective
Telmisartan (160) for 14 days *vs.* standard treatment	Duarte 72††	Not specified	Argentina	May 14-Jul 30	41 - 41	HR for discharge: 2.02 (95% CI 1.14 to 3.59) Time to discharge from the hospital: 9 *vs.* 15 days (*P* = 0.0124) 30-day mortality: 2/38 (5%) *vs.* 4/34 (12%) (*P* = 0.41) Serum CRP levels on day 5: 24.2 (SD 31.4) *vs.* 51.1 (44.8) mg/L (*P* < 0.05).	Effective
Enoxaparin (0.75-2 mg/kg for 4-14 days) vs. enoxaparin (40 or 80) or unfractionated heparin (15,000-22,500 IU)†††	Lemos 75	Severe and intubated	Brazil	Apr-Jul	10 - 10	Successful liberation from MV by day 28: 8 (80%) *vs*. 3 (30%) (HR 4.0, 95% CI 1.04 to 15.05, *P* = 0.031) Ventilator-free days: 15 6-16 *vs*. 0 [0-11] days (*P* = 0.028)]; 28-day mortality: 1 (10%) *vs*. 3 (30%) (*P* = 0.264).	Effective
Calcifediol (0.532 on day 1; 0.266 on day 3 and 7, then weekly) *vs.* standard treatment [hydroxychloroquine, azithromycin]	Entrenas Castillo 78	Not specified	Spain	Not specified	50 - 26	ICU admission: 1 (2%) *vs.* 13 (50%) (adjusted OR 0.03, 95% CI 0.003 to 0.25).	Effective
CM4620-IE (Auxora: calcium release-activated calcium channel inhibitor) (2.0 mg/kg/day continuous infusion on day 1; 1.6 mg/kg/day on day 2-3) *vs.* standard treatment.	Miller 79	Severe/ critical	The US	Apr 8-May 13	20 - 10	IMV or death by day 30: 3/17 (18%) *vs.* 5/9 (56%) in participants with severe COVID-19 (HR 0.23, 95% CI 0.05 to 0.96; *P* < 0.05) The mean difference in 8-point ordinal scale between groups was statistically significant at day 6 and day 9-12 (*P* < 0.05).	Effective
Ruxolitinib (Janus-associated kinase inhibitors) (10) *vs.* standard treatment	Cao 81	Severe	China	Feb 9-Feb 28	20 - 21	Improvement of chest CT scans on day 14: 18 (90%) *vs.* 13 (62%) (*P* = 0.0495) 28-day mortality: 0 (0%) *vs.* 3 (14%) (*P* = 0.232) Time to clinical improvement (a 2-point reduction on a 7-category ordinal scale or discharge from hospital): 12 10-19 *vs.* 15 10-18 days (*P* = 0.147) (HR 1.669, 95% CI 0.836 to 3.335) Time to lymphocyte recovery: 5 2-7 *vs.* 8 2-11 days (*P* = 0.033).	Effective
Leflunomide (DHODH inhibitor) (100 day 1-3; 20 day 4-10) *vs.* standard treatment [Umifenovir].	Hu 82	Moderate	China	Feb 20-Feb 28	5 -5	Duration of viral shedding: 5 *vs.* 11 days (*P* = 0.046) The difference in the level of serum CRP measured before treatment and on day: 32 [5.6-not tested] *vs.*0 [0-9.1] mg/L (*P* = 0.047).	Possible effect
***Immunotherapy***							
**Interferon**							
IFN-β1a (12 million IU 3 times weekly for 2 weeks) *vs.* standard treatment [Hydroxychloroquine plus lopinavir/ritonavir or atazanavir/ritonavir].	Davoudi-Monfared 83	Severe	Iran	Feb 29-Apr 3	42 - 39	28-day mortality: 19% *vs.* 44% (*P* = 0.015) Rate of discharge from the hospital: 67% *vs.* 44% (OR 2.5, 95% CI 1.05 to 6.37) Early administration of IFN-β1a reduced mortality (OR 13.5, 95% CI 1.5 to 118). Time to clinical improvement: 9.7 ± 5.8 *vs.* 8.3± 4.9 days (*P* = 0.95).	Effective
IFN-β1b (3 doses of 8 million IU on alternate days) plus ribavirin (800) for 14 days *vs.* standard treatment [Lopinavir/ritonavir]	Hung 44	Mild/ moderate	Hong Kong	Feb 10-Mar 20	86 - 41	Time to a NEWS2 of 0: 4 3-8 *vs.* 8 7-9 days (HR 3.92, 95% CI 1.66 to 9.23) Time to a SOFA score of 0: 3.0 [1.0-8.0] *vs.* 8.0 [6.5-9.0] days (HR 1.89, 95% CI 1.03 to 3.49) Length of hospital stay: 9.0 [7.0-13.0] *vs.* 14.5 [9.3-16.0] days (HR 2.72, 95% CI 1.2 to 6.13) Time to viral clearance: 7 5-11 *vs.* 12 8-15 days (HR 4.37, 95% CI 1.86 to 10.24, *P* = 0.001).	Effective
IFN-β1b (250 mcg on alternate days for 2 weeks) *vs.* standard treatment	Rahmani 84	Severe	Iran	Apr 20-May 20	33- 33	Discharge from hospital by day 14: 26 (79%) *vs.* 18 (55%) (OR 3.09, 95% CI 1.05 to 9.11, *P* = 0.03) ICU admission: 14 (42%) *vs.* 22 (67%) (*P* = 0.04) Time to clinical improvement (a 2-point reduction on a 6-category ordinal scale): 9 6-10 *vs.* 11 9-15 days (*P* = 0.002).	Effective
Inhaled IFN-κ (2) plus TFF2 (5) for 6 days *vs.* standard treatment	Fu 85	Moderate	China	Mar 23-May 23	40 - 40	Time to improvement of chest CT: 6.2 (95% CI 5.1-7.3) *vs.* 8.8 (95% CI 7.6-10.0) days (*P* = 0.002) Time to viral clearance: 3.8 (95% CI 2.1-5.5) *vs.* 7.4 (95% CI 4.6-10.2) days (*P* = 0.031).	Effective
A: Novaferon (40 mcg) B: Novaferon and lopinavir (800)/ritonavir (200) C: Lopinavir/ritonavir.	Zheng 86	Moderate/ severe	China	Feb 1-Feb 20	30 (A) 30 (B) 29 (C)	Viral clearance on day 6: 15/30 (A, 50%; *P* = 0.04) or 18/30 (B, 60%; *P* = 0.0053) *vs.* 7/29 (C, 24%) Time to viral clearance: 6 (A, *P* = 0.417) or 6 (B, *P* = 0.036) *vs.* 9 (C) days.	Possible effect
**Convalescent plasma**							
4-13 mL/kg *vs.* standard treatment	Li 87	Severe/ life-threatening	China	Feb 14-Apr 1	52 - 51	Clinical improvement (a 2-point reduction on a 6-category ordinal scale or discharge from hospital) within 28 days: 27 (52%) *vs.* 22 (43%) (HR 1.40, 95% CI 0.79 to 2.49) Clinical improvement within 28 days for the participants with severe COVID-19: 21/23 (91%) *vs.* 15/22 (28%) (HR 2.15, 95% CI 1.07 to 4.32) 28-day mortality: 8 (16%) *vs.* 12 (24%) (OR 0.59, 0.22 to 1.59) Viral clearance within 72 hours: 41 (87%) *vs.* 15 (38%) (OR 11.39, 95% CI 3.91 to 33.18).	Effective (severe COVID-19 subgroup)
200 mL (day1-2) *vs.* standard treatment	Agarwal 89††	Moderate	India	Apr 22-Jul 14	235 - 229	28-day mortality: 34 (15%) *vs.* 31 (14%) (adjusted OR 1.06, 95% CI 0.61 to 1.83) Disease progression (PaO_2_/FiO_2_ < 100): 44 (19%) *vs.* 41 (18%) (adjusted OR 1.09, 95% CI 0.67 to 1.77).	Not effective
300 mL *vs.* standard treatment	Gharbharan 184††	Not specified	Nether-lands	Apr 8-Jun 10	43 - 43	60-day mortality: 6 (14%) *vs.* 11 (26%) (OR 0.95, 95% CI 0.20 to 4.67) Improvement in WHO-OSCI on day 15: 25 (58%) *vs.* 25 (58%) (OR 1.30, 95% CI 0.52 to 3.32).	Not effective
250-300 mL *vs.* standard treatment	Avendaño-Solà 88††	Not specified	Spain	Apr 4-Jul 10	38 - 43	Initiation of MV or death by day 15: 0 (0%) *vs.* 6 (14%) (*P* = 0.03) 28-day mortality: 0 (0%) *vs.* 4 (9%) (*P* = 0.06).	Effective
200 mL (day1-2) *vs.* deferred treatment‡‡‡	Barcells 185††	At risk for progression	Chile	May 10-Jul 18	28 - 30	A composite of MV, hospitalization for > 14 days, or death: 9 (32%) *vs.* 10 (33%) (OR 0.95, 95% CI 0.32 to 2.84) 13 participants (43%) from the deferred group received convalescent plasma based on clinical aggravation.	Not effective
**Other immunotherapies**							
rhG-CSF 5 mcg/kg (day 1-3)* vs.* standard treatment	Cheng 90	Lympho-penia	China	Feb 18-Apr 10	100 - 100	21-day mortality: 2 (2%) *vs.* 10 (10%) (HR 0.19, 95% CI 0.04 to 0.88) Disease progression§§§: 2 (2%) *vs.* 15 (15%) (difference -13%, 95% CI -21.4% to -5.4%) Time to clinical improvement (a 1-point reduction on a 7-category ordinal scale or discharge from hospital): 12 10-16 *vs.* 13 11-17 (HR 1.28, 95% CI 0. 95-1.71, *P* = 0.06).	Effective (lympho-penia)
Intravenous immunoglobulin 0.5g/kg/day for 3 days plus methylprednisolone (40 mg once) *vs.* standard treatment	Sakoulas 91††	Moderate/severe (except patients with MV)	The US	May 1-Jun 16	16 - 17	(Among subjects with alveolar-arterial oxygen gradient of >200 mmHg at enrollment) Initiation of MV within 30 days: 2/14 (14%) *vs.* 7/12 (58%) (*P* = 0.038), Length of hospital stay: 11 (range 5-22) *vs.* 19 (4-30) days (*P* = 0.013) Length of ICU stay: 2.5 (range 0-16) *vs.* 12.5 (1-29) days (*P* = 0.006) Difference in PaO_2_/FiO_2_ on day 7: +131 (+35 to +330) *vs.*+44.5 (-115 to +157) (*P* = 0.01).	Effective
Vilobelimab (anti-C5a antibody IFX-1) 800 mg (day 1, 2, 4, 8, and 15) *vs.* standard treatment	Vlaar 92	Severe	Nether-lands	Mar 31-Apr24	15 - 15	28-day mortality: 2 (13%) *vs.* 4 (27%) (adjusted HR 0.65, 95% CI 0.10 to 4.14) Difference in the change in PaO_2_/FiO_2_on day 5 (least squares mean): 17% (SD 63) *vs.* 41% (difference -24%, 95% CI -58% to 9%,* P* = 0.15).	Not effective
CIGB-325 (anti-CK2) 2.5 mg/kg (day 1-5) *vs.* standard treatment	Cruz 93††	Not specified	Cuba	Jun 1-Jun 16	10 - 10	Reduction in the number of pulmonary lesions on the chest CT: 5/6 (83%) *vs*. 3/7 (43%) (Bayesian *P* (difference > 0) = 0.951). Time to viral clearance: 11 (SD 8) *vs*. 12 (SD 6) days (*P* = 0.614).	Effective

CI: confidence interval; COVID-19: coronavirus disease 2019; CRP: C-reactive protein; CT: computed tomography; DHODH: dihydroorotate dehydrogenase; HR: hazard ratio; ICU: intensive care unit; IFN: interferon; IMV: invasive mechanical ventilation; IQR: interquartile range; IU: international unit; MV: mechanical ventilation; NEWS2: National Early Warning Score 2; OSCI: ordinal scale for clinical improvement; OR: odds ratio; PCR: polymerase chain reaction; RCT: randomized controlled trial; rhG-CSF: Recombinant human granulocyte colony-stimulating factor; RR: relative risk; SD: standard deviation; SE: standard error; SOFA: sequential organ failure assessment; WHO: World Health Organization. All of the presented studies were conducted in 2020. In the outcome description, the former is the data of the treatment group and the latter is the data of the control group. *The United States, Denmark, the United Kingdom, Greece, Germany, South Korea, Mexico, Spain, Japan, and Singapore. †The United States, Italy, Spain, Germany, Hong Kong, Singapore, South Korea, and Taiwan. ‡750 mg for participants with a mild or ordinary condition or 1,000 mg for participants with a severe or critical condition. §Defined as normalization of body temperature, respiratory rate, oxygen saturation, cough, and absorption of pulmonary infection on chest CT. ¶Resolving from fever to an axillary temperature of 36.6°C or below, normalization of SpO_2_ (> 94% on room air), and disappearance of respiratory symptoms including nasal congestion, cough, sore throat, sputum production, and shortness of breath. **The most common adverse event in the treatment group was diarrhea (7/70). ††Preprints from Medrxiv.org. ‡‡Defined as development of fever higher than 101 F for more than 72 hours, shortness of breath by minimal exertion (10-Step walk test), derangement of basic laboratory parameters (absolute lymphocyte count < 1,000 mm^3^ or raised serum C-reactive protein level), or appearance of infiltrates on chest radiograph during course of treatment. §§In the case of azithromycin coadministration, a single 1.0-mg loading dose of colchicine was administered. ¶¶Chloroquine or hydroxychloroquine was administered to 100% and 96% of participants in the treatment and the control group, respectively. Azithromycin was administered to 93% and 92% of participants in the treatment and the control group, respectively. ***Defined as a Borg score > 3, improved dyspnea, stopped fever for 72 hours, SO_2_ > 93%, tolerated oral regimen, normal urinary output, and reduced C-reactive protein level without any side effects. †††The dosage was determined according to age, body weight, and creatinine clearance. ‡‡‡The deferred treatment group received convalescent plasma only when a PaO_2_/FiO_2_ < 200 criterion was met during hospitalization or when the patient still required hospitalization for symptomatic COVID-19 more than 7 days after enrollment. §§§Progression to acute respiratory distress syndrome, sepsis, or septic shock.

**Table 4:** Summary of meta-analyses evaluating therapeutics for COVID-19

Comparisons	First author	No. of studies		No. of participants		Type of metrics	Model	Summary effect (95% CI)	*P*	*I*^2^ (*P*)	Publication bias	Conclusion
***Antiviral agents***												
**Remdesivir**												
Mortality	Misra 22	2 20, 21	0	54 (54)/696 (696)	64 (64)/599 (599)	RR	Random	0.74 (0.40 to 1.37)	NA	58% (0.12)	NA	Not effective
Clinical recovery	Misra 22	2 20, 21	0	437 (437)/696 (696)	318 (318)/599 (599)	RR	Fixed	1.17 (1.07 to 1.29)	NA	0% (0.70)	NA	Effective
Clinical improvement* (5 *vs.* 10-days of treatment)	Jiang 26	2 24, 25	0	263 (263)/391 (391)	233 (233)/390 (390)	OR	Random	1.33 (1.01 to 1.76)	NA	NA	NA	Favors 5-day treatment
Adverse events	Misra 22	2 20, 21	0	258 (258)/696 (696)	222 (222)/599 (599)	RR	Fixed	0.91 (0.79 to 1.05)	NA	7% (0.30)	NA	Inconclusive
Serious adverse events	Juul 186	2 20, 21	0	142 (142)/ 554 (554)	161 (161)/438 (438)	RR	Random	0.77 (0.63 to 0.94)	0.01	0.0% (0.66)	NA	Inconclusive
**Favipiravir**												
Clinical improvement by day 14	Shrestha 187	2 31, 188	1	73 (41)/84 (49)	49 (21)/75 (30)	RR	Fixed	1.29 (1.08 to 1.54)	0.005	16% (0.30)	NA	Effective
	Shrestha 187	2 31, 188	0	41 (41)/49 (49)	21 (21)/30 (30)	RR	Fixed	1.12 (0.87 to 1.44)	0.37	0% (0.98)	NA	Not effective
Viral clearance by day 14	Shrestha 187	2 31, 188	1	77 (44)/84 (49)	61 (28)/75 (30)	RR	Random	1.06 (0.84 to 1.33)	0.65	67% (0.05)	NA	Inconclusive
	Shrestha 187	2 31, 188	0	44 (44)/49 (49)	28 (28)/30 (30)	RR	Random	0.95 (0.74 to 1.22)	0.67	41% (0.19)	NA	Inconclusive
**Umifenovir**												
Clinical recovery	Misra 22	1 34	1	51 (32)/69 (35)	40 (13)/65 (17)	RR	Fixed	1.08 (0.85 to 1.38)	NA	0% (0.42)	N	Not effective
Viral clearance (on day 14)	Huang 35	1 34	4	122 (32)/140 (35)	174 (13)/247 (17)	RR	Random	1.27 (1.04 to 1.55)	0.02	63% (0.03)	NA	Possible effect
Adverse events	Misra 22	1 34	1	8 (5)/69 (35)	4 (0)/65 (17)	RR	Fixed	1.80 (0.52 to 6.19)	NA	10% (0.29)	N	Inconclusive
**Lopinavir/ritonavir**												
Clinical recovery	Misra 22	2 34, 40	1	135 (107)/185 (133)	110 (83)/165 (117)	RR	Fixed	1.08 (0.94 to 1.24)	NA	0% (0.70)	N	Not effective
Viral clearance	Wang 42	2 34, 40	1	96 (61)/153 (93)	141 (53)/209 (88)	RR	Fixed	0.90 (0.76 to 1.07)	0.225	33.9% (0.220)	N	Inconclusive
	Liu 43	2 34, 40	0	48 (48)/80 (80)	45 (45)/78 (78)	RR	Random	0.99 (0.76 to 1.29)	0.93	0% (0.74)	NA	Inconclusive
Adverse events	Misra 22	2 34, 40	1	67 (58)/185 (133)	53 (49)/165 (117)	RR	Random	1.73 (0.57 to 5.26)	NA	67% (0.05)	N	Inconclusive
Increased serum creatinine	Zhong 189	1 40	1	4 (2)/147 (95)	7 (7)/147 (99)	RR	Random	0.86 (0.66 to 11.97)	NA	61% (0.110)	NA	Inconclusive
**Hydroxychloroquine**												
28-day mortality	Elsawah 190	2 179, 180	0	0 (0)/239 (239)	0 (0) 264 (264)	RD	Fixed	0.00 (-0.01 to 0.01)	1.00	0% (1.00)	NA	Not effective
Mortality	Yang 191	1 192	4	91 (0)/451 (15)	284 (0)/930 (15)	OR	Random	1.23 (0.38 to 3.97)	0.73	88% (<0.0001)	N	Not effective
	Das 193	0	8	268 (0)/2009 (0)	533 (0)/3671 (0)	OR	Random	0.87 (0.46 to 1.64)	0.66	92% (<0.00001)	Y	Not effective
	Thoguluva Chandrasekar 194	0	4	452 (0)/2111 (0)	125 (0)/1041 (0)	OR	Random	1.86 (1.38 to 2.50)	<0.001	29% (0.234)	NA	Harmful
	Zang 195	0	3	63 (0)/311 (0)	27 (0)/268 (0)	RR	Fixed	1.92 (1.26 to 2.93)	0.003	0% (0.508)	NA	Harmful
Deterioration†	Yang 191	3 55, 180, 192	3	48 (2)/494 (116)	29 (4)/540 (126)	OR	Random	2.46 (0.42 to 14.45)	0.32	69% (0.007)	N	Not effective
	Liu 43	3 55, 180, 192	0	2 (2)/115 (115)	4 (4)/125 (125)	RR	Random	0.96 (0.10 to 9.66)	0.98	41% (0.8)	NA	Not effective
	Wang 42	2 55, 192	3	244 (1)/843 (46)	858 (4)/4112 (46)	RR	Random	1.05 (0.61 to 1.81)	NA	62.5% (0.031)	N	Not effective
Clinical progression within 5-7 days‡	Elsawah 190	2 55, 192	2	11 (1)/89 (46)	6 (4)/83 (46)	RD	Fixed	0.06 (-0.03 to 0.15)	0.18	76% (0.006)	NA	Not effective
Clinical progression within 28 days‡	Elsawah 190	2 179, 180	0	9 (9)/206 (206)	11 (11)/234 (234)	RD	Fixed	-0.00 (-0.04 to 0.04)	0.86	0% (0.33)	NA	Not effective
Death or invasive MV	Putman 196	0	2	166 (0)/895 (0)	83 (0)/654 (0)	HR	Random	1.03 (0.82 to 1.29)	0.81	0% (0.75)	NA	Not effective
Death or deterioration†	Sarma 197	2 55, 192	1	5 (1)/66 (46)	4 (4)/62 (46)	OR	Random	1.37 (0.09 to 21.97)	0.82	59% (0.09)	NA	Not effective
Death or deterioration† (≤ 400 mg/day)	Yang 191	2 55, 192	2	64 (1)/365 (46)	270 (4)/787 (46)	OR	Random	0.64 (0.14 to 2.81)	0.55	84% (0.0002)	N	Not effective
Death or deterioration† (> 400 mg/day)	Yang 191	1 180	1	5 (1)/90 (70)	0 (0)/96 (80)	OR	Fixed	6.17 (0.71 to 53.47)	0.10	0% (0.67)	N	Not effective
Clinical recovery	Misra 22	2 180, 192	5	1026 (69)/1474 (90)	1054 (67)/1376 (90)	RR	Random	0.93 (0.84 to 1.04)	NA	74% (<0.01)	Y	Not effective
	Talaie 198	2 55, 180	0	70 (70)/106 (106)	67 (67 )/106 (106)	RR	Random	1.04 (0.85 to 1.28)	NA	79.3% (0.028)	NA	Not effective
Radiological improvement	Ullah 199	2 55, 192	1	40 (30)/56 (46)	33 (24)/58 (46)	OR	Random	1.98 (0.47 to 8.36)	0.36	54% (0.11)	N	Not effective
Radiological progression	Sarma 197	2 55, 192	0	7 (7)/46 (46)	16 (16)/46 (46)	OR	Random	0.31 (0.11 to 0.90)	0.03	16% (0.27)	NA	Not effective
Viral clearance	Singh 200	2 180, 192	1	80 (72)/99 (85)	81 (79)/111 (95)	RR	Random	1.05 (0.79 to 1.38)	0.744	62% (0.07)	Y	Inconclusive
	Liu 43	2 180, 192	0	77 (77)/90 (90)	80 (80)/90 (90)	RR	Random	0.98 (0.89 to 1.07)	0.65	0% (0.54)	NA	Inconclusive
	Elavarasi 201	0	3	217 (0)/240 (0)	152 (0)/203 (0)	RR	Random	1.21 (0.64 to 2.29)	0.56	87% (0.0006)	NA	Inconclusive
Adverse events	Wang 42	3 55, 180, 192	1	35 (27)/200 (116)	10 (10)/223 (126)	RR	Fixed	3.62 (1.93 to 6.79)	NA	17.6% (0.303)	N	Possible harm
	Zhong 189	3 55, 180, 192	0	27 (27)/116 (116)	10 (10)/126 (126)	RR	Random	2.75 (1.42 to 5.33)	NA	0% (0.442)	NA	Possible harm
Adverse events (gastrointestinal)	Elsawah 190	3 179, 180, 192	0	157 (157)/254 (254)	7 (7)/279 (279)	RD	Fixed	0.59 (0.55 to 0.64)	<0.00001	99% (<0.00001)	NA	Possible harm
Adverse events (CNS)	Elsawah 190	3 55, 179, 180	0	65 (65)/270 (270)	3 (3)/295 (295)	RD	Fixed	0.23 (0.18 to 0.28)	<0.00001	99% (<0.00001)	NA	Possible harm
Adverse events (neurological)	Ullah 199	2 180, 192	1	2 (2)/111 (101)	2 (0)/123 (111)	OR	Random	1.26 (0.20 to 7.98)	0.81	0% (0.37)	N	Inconclusive
Adverse events (cardiac)	Elsawah 190	2 179, 180	0	3 (3)/239 (239)	0 (0)/264 (264)	RD	Fixed	0.01 (-0.01 to 0.03)	0.16	84% (0.01)	NA	Inconclusive
**Hydroxychloroquine plus azithromycin**												
Mortality	Das 193	0	4	NA (0)/1145 (0)	NA (0)/1165 (0)	OR	Random	2.84 (2.19 to 3.69)	<0.00001	0% (0.43)	Y	Harmful
	Yang 191	0	3	214 (0)/854 (0)	46 (0)/395 (0)	OR	Fixed	2.34 (1.63 to 3.36)	<0.00001	0% (0.85)	N	Harmful
Deterioration†	Yang 191	0	3	101 (0)/840 (0)	25 (0)/414 (0)	OR	Random	4.97 (0.01 to 4781.7)	0.65	95% (<0.00001)	N	Not effective
	Wang 42	0	2	115 (0)/328 (0)	833 (0)/3969 (0)	RR	Random	0.93 (0.17 to 5.09)	NA	94.2% (<0.001)	N	Not effective
**Corticosteroids**												
Mortality	Lu 67	0	4	94 (0)/329 (0)	58 (0)/408 (0)	RR	Random	2.00 (0.69 to 5.75)	NA	90% (<0.001)	NA	Not effective
Mortality (severe COVID-19 subgroup)	Ye 68	0	2	NA (0)/227 (0)	NA (0)/104 (0)	HR	Random	2.30 (1.00 to 5.29)	NA	0% (0.768)	NA	Not effective
Time to viral clearance	Sarkar 69	0	2	82	69	MD	Random	1.42 (-0.52 to 3.37)	0.15	0% (0.52)	NA	Not effective
**Renin-angiotensin-aldosterone system inhibitors for patients with hypertension**												
Mortality (ACEI)	Pranata 73	0	3	29 (0)/110 (0)	87 (0)/326 (0)	OR	Random	0.68 (0.39 to 1.17)	0.16	0% (0.62)	Y	Not effective
Mortality (ARB)	Pranata 73	0	3	29 (0)/158 (0)	87 (0)/326 (0)	OR	Random	0.51 (0.29 to 0.90)	0.02	22% (0.28)	Y	Effective
Mortality (ACEI or ARB)	Flacco 74	0	4	NA (0)/921 (0)	NA (0)/1491 (0)	OR	Random	0.88 (0.68 to 1.14)	0.33	24% (0.27)	N	Not effective
**Anticoagulants**												
Mortality	Lu 76	0	5	536 (0 )/2886 (0)	947 (0)/5647 (0)	RR	Random	0.86 (0.69 to 1.09)	0.218	47.4% (0.107)	NA	Not effective
Heparin - mortality (severe COVID-19 subgroup)	Abdel-Maboud 77	0	2	50 (0)/126 (0)	115 (0)/368 (0)	RR	Random	1.09 (0.84 to 1.42)	NA	0% (0.537)	NA	Not effective
**Convalescent plasma**												
Mortality	Talaie 198	1 87	2	10 (8)/82 (52)	21 (12)/81 (51)	RR	Random	(0.26 to 1.03)	NA	0% (0.484)	N	Not effective
Clinical improvement	Talaie 198	1 87	2	46 (27)/82 (52)	32 (22)/81 (51)	RR	Random	1.41 (1.01 to 1.98)	NA	66.6% (0.050)	Y	Effective
Viral clearance	Sarkar 202	1 87	2	54 (41)/68 (52)	18 (15)/76 (51)	OR	Random	11.29 (4.92 to 25.92)	<0.00001	0% (0.40)	Y	Possible effect
**Tocilizumab**												
Mortality	Lan 203§	0	7	39 (0)/241 (0)	85 (0)/352 (0)	RR	Random	0.61 (0.31 to 1.22)	0.16	68% (0.005)	NA	Not effective
Mortality (lopinavir/ritonavir subgroup)¶	Malgie 94	0	2	7 (0)/94 (0)	22 (0)/56 (0)	RD	Random	-0.31 (-0.57 to -0.05)	NA	NA	Y	Effective
ICU admission and initiation of MV	Lan 203§	0	5	47 (0)/134 (0)	44 (0)/279 (0)	RR	Random	1.51 (0.33 to 6.78)	0.59	86% (<0.00001)	NA	Not effective

ACEI: angiotensin-converting enzyme inhibitor; ARB: angiotensin receptor blocker; ARDS: acute respiratory distress syndrome; CI: confidence interval; CNS: central nervous system; COVID-19: coronavirus disease 2019; ECG: electrocardiogram; HR: hazard ratio; ICU: intensive care unit; MD: mean difference; MV: mechanical ventilation; NA: not applicable; OR: odds ratio; RCT: randomized controlled trial; RD: risk difference; RR: relative risk. The RCTs included in the meta-analyses of this table were also included in our target RCTs and are presented in **Table 3**, except an RCT 25 with no peer-reviewed or preprint report released, an RCT 192 published in Chinese, and an RCT 188 in which a statistical analysis was not conducted. *A 2-point reduction on a 7-category ordinal scale. †Progression to severe COVID-19. ‡An increase in severity compared to the baseline severity. §One participant in the treatment arm of one included study was diagnosed as suspected COVID-19 with a negative PCR result. ¶All participants received lopinavir plus ritonavir but did not receive corticosteroids.

## Abbreviations

ACEI: angiotensin-converting enzyme inhibitor
ACE2: angiotensin-converting enzyme 2
ARB: angiotensin receptor blocker
ARDS: acute respiratory distress syndrome
CI: confidence interval
COVID-19: coronavirus disease 2019
CRAC: calcium release-activated calcium
CT: computed tomography
EC_50_: 50% maximal effective concentration
ExoN: exoribonuclease
FDA: Food and Drug Administration
HCV: hepatitis C virus
HIV: human immunodeficiency virus
HR: hazard ratio
ICU: intensive care unit
IFN: interferon
IL: interleukin
IQR: interquartile range
MERS: Middle East respiratory syndrome
MV: mechanical ventilation
NEWS2: National Early Warning Score 2
OR: odds ratio
PCR: polymerase chain reaction
PICO: Participants, Interventions, Comparisons, and Outcomes
PRISMA: Preferred Reporting Items for Systematic Reviews and Meta-Analyses
RCT: randomized controlled trial
RdRp: RNA dependent RNA polymerase
rhG-CSF: recombinant human granulocyte colony-stimulating factor
RR: relative risk
SARS: severe acute respiratory syndrome
SARS-CoV-2: severe acute respiratory syndrome coronavirus 2
SD: standard deviation
SI: selectivity index
SOFA: sequential organ failure assessment
WHO: World Health Organization
