# Supplementary material for: Treatment of severe acute respiratory syndrome (SARS), Middle East respiratory syndrome (MERS), and coronavirus disease 2019 (COVID-19): a systematic review of in vitro, in vivo, and clinical trials
Source: Theranostics. 2021 Jan 1;11(3):1207–31. doi: 10.7150/thno.48342 (PMC7738873; doi:10.7150/thno.48342)
Supplement: Supplementary file 1 — Supplementary table S1. [file thnov11p1207s1.pdf]

Supplementary Material

Treatment of severe acute respiratory syndrome (SARS), Middle East respiratory syndrome (MERS), and coronavirus disease 2019 (COVID-19): a systematic review of *in vitro*, *in vivo*, and clinical trials. Young Joo Han, Keum Hwa Lee, Sojung Yoon, Seoung Wan Nam, Seohyun Ryu, Dawon Seong, Jae Seok Kim, Jun Young Lee, Jae Won Yang, Jinhee Lee, Ai Koyanagi, Sung Hwi Hong, Elena Dragioti, Joaquim Radua, Lee Smith, Hans Oh, Ramy Abou Ghayda, Andreas Kronbichler, Maria Effenberger, Daniela Kresse, Sara Denicolò, Woosun Kang, Louis Jacob, Hanwul Shin, and Jae Il Shin

Table S1. Therapeutic agents that showed no potent effects against SARS-CoV-2 in *in vitro* studies.

Therapeutics	First author	Findings
**Antiviral agents**		
Ribavirin	Wang [1]	EC_{50} = 109.5 μM; CC_{50} > 400 μM; SI > 3.65
	Choy [2]	EC_{50} > 500 μM; CC_{50} > 100 μM
Lopinavir	Choy [2]	EC_{50} = 26.1 μM; CC_{50} > 49.75 μM
Ritonavir	Choy [2]	EC_{50} > 100 μM; CC_{50} > 48.91 μM
Favipiravir	Wang [1]	EC_{50} = 61.88 μM; CC_{50} > 400 μM; SI > 6.46
	Choy [2]	EC_{50} > 100 μM; CC_{50} > 100 μM
Oseltamivir	Choy [2]	EC_{50}>100 μM; CC_{50} > 100 μM
	Wang [3]	Not effective
Galidesivir	Choy [2]	EC_{50} > 100 μM; CC_{50} > 100 μM
Tenofovir	Choy [2]	Not effective
Baloxavir	Choy [2]	EC_{50} > 100 μM; CC_{50} = 85.9 μM
	Wang [3]	Partial inhibition (up to 29%) at a high concentration of 50 μM
Laninamivir	Wang [3]	Not effective
Peramivir	Wang [3]	Not effective
Zanamivir	Wang [3]	Not effective
R-1479	Choy [2]	Not effective
**Antibacterial agents**		
Oritavancin	Choy [2]	Not effective
Dalbavancin	Choy [2]	Not effective
**Other agents**		
Nafamostat	Wang [1]	EC_{50} = 22.5 μM; CC_{50} > 100 μM; SI > 4.44
Chlorpromazine	Choy [2]	Not effective
Fludarabine	Choy [2]	Not effective

CC_{50}: 50% cytotoxic concentration; COVID-19: coronavirus disease 2019; EC_{50}: 50% maximal effective concentration; SI: selectivity index.

References

1. Wang M, Cao R, Zhang L, Yang X, Liu J, Xu M, et al. Remdesivir and chloroquine effectively inhibit the recently emerged novel coronavirus (2019-nCoV) in vitro. Cell Res. 2020; 30: 269-71.
2. Choy KT, Wong AY, Kaewpreedee P, Sia SF, Chen D, Hui KPY, et al. Remdesivir, lopinavir, emetine, and homoharringtonine inhibit SARS-CoV-2 replication in vitro. Antiviral Res. 2020; 178: 104786.
3. Wang X, Cao R, Zhang H, Liu J, Xu M, Hu H, et al. The anti-influenza virus drug, arbidol is an efficient inhibitor of SARS-CoV-2 in vitro. Cell Discov. 2020; 6: 28.
